# Missing binary outcomes under covariate‐dependent missingness in cluster randomised trials

**DOI:** 10.1002/sim.7334

**Published:** 2017-05-29

**Authors:** Anower Hossain, Karla DiazOrdaz, Jonathan W. Bartlett

**Affiliations:** ^1^ Department of Medical Statistics London School of Hygiene and Tropical Medicine London U.K.; ^2^ Institute of Statistical Research and Training (ISRT) University of Dhaka Dhaka 1000 Bangladesh; ^3^ Statistical Innovation Group AstraZeneca Cambridge U.K.

**Keywords:** cluster randomised trials, missing binary outcome, baseline covariate‐dependent missingness, complete records analysis, multiple imputation

## Abstract

Missing outcomes are a commonly occurring problem for cluster randomised trials, which can lead to biased and inefficient inference if ignored or handled inappropriately. Two approaches for analysing such trials are cluster‐level analysis and individual‐level analysis. In this study, we assessed the performance of unadjusted cluster‐level analysis, baseline covariate‐adjusted cluster‐level analysis, random effects logistic regression and generalised estimating equations when binary outcomes are missing under a baseline covariate‐dependent missingness mechanism. Missing outcomes were handled using complete records analysis and multilevel multiple imputation. We analytically show that cluster‐level analyses for estimating risk ratio using complete records are valid if the true data generating model has log link and the intervention groups have the same missingness mechanism and the same covariate effect in the outcome model. We performed a simulation study considering four different scenarios, depending on whether the missingness mechanisms are the same or different between the intervention groups and whether there is an interaction between intervention group and baseline covariate in the outcome model. On the basis of the simulation study and analytical results, we give guidance on the conditions under which each approach is valid. © 2017 The Authors. *Statistics in Medicine* Published by John Wiley & Sons Ltd.

## 1 Introduction

Cluster randomised trials (CRTs), also known as group randomised trials, are increasingly being used to evaluate the effectiveness of interventions in health services research 1, 2. The unit of randomisation for such trials are identifiable clusters of individuals such as medical practices, schools or entire communities. However, individual‐level outcomes of interest are observed within each cluster. One important feature of CRTs is that the outcomes of individuals within the same cluster are more likely to be similar to each other than those from different clusters, which is usually quantified by the intraclass correlation coefficient (ICC, denoted as *ρ*). Although typically in primary care and health research the value of ICC is small (0.001<*ρ*<0.05) 3, it can lead to substantial variance inflation factors and should not be ignored 2, 4. This is because ignoring the dependence of the outcomes of individuals within the clusters will underestimate the variance of the intervention effect estimates and consequently give inflated type I error rates 5. It is well known that the power and precision of CRTs are lower compared with trials that individually randomise the same number of units 2. However, in practice, CRTs have several advantages including that the nature of the intervention itself may dictate its application at the cluster level, less risk of intervention contamination and administrative convenience 6. These advantages are sometimes judged by researchers to outweigh the potential loss of statistical power and precision.

Missing data are a commonly occurring threat to the validity and efficiency of CRTs. In a systematic review of CRTs published in English in 2011, 72% of trials had missing values either in outcomes or in covariates or in both, and only 34% of them reported how missing data had been handled 7. Dealing with missing data in CRTs is complicated because of the clustering of the data. In statistical analysis, if there are missing values, an assumption must be made about the relationship between the probability of data being missing and the underlying values of the variables involved in the analysis. The mechanisms that caused the data to be missing can be classified into three broad categories. Data are missing completely at random (MCAR) if the probability of missingness is independent of the observed and unobserved data. MCAR is generally a very restrictive assumption and is unlikely to hold in many studies. A more plausible assumption is missing at random (MAR) where, conditioning on the observed data, the probability of missingness is independent of the unobserved data. Missing not at random is the situation where the probability of missingness depends on both the observed and unobserved data. In CRTs, an assumption regarding missing outcomes that is sometimes plausible is that missingness depends on baseline covariates, but conditioning on these baseline covariates, not on the outcome itself. We refer to this as covariate‐dependent missingness (CDM). This is an example of MAR when baseline covariates are fully observed. In this paper, we will consider the case of a binary outcome that is partially observed and assume that all baseline covariates are fully observed.

Two approaches for analysing CRTs are cluster‐level analyses, which derive summary statistics for each cluster, and individual‐level analyses, which use the data for each individual in each cluster 6. Complete records analysis (CRA) and multiple imputation (MI) (described in Section 3) are the most commonly used methods for handling missing data. A number of recent studies have investigated how to handle missing binary outcomes in CRTs under the assumption of CDM 8, 9, 10, 11. However, as we describe in detail in Section 3, these previous studies simulated datasets in ways that arguably do not correspond to how data arise in CRTs raising doubt about their conclusions.

In the case of missing outcome under MAR for individually randomised trials, Groenwold *et al.* 12 showed that CRA with covariate adjustment and MI give similar estimates as long as the same covariates and same functional form are used. It can be anticipated that a similar result holds for CRTs. In the case of missing continuous outcomes in CRTs, Hossain *et al.* 13 showed that there is no gain in terms of bias or efficiency of the estimates using MI over CRA adjusted for covariates, where both approaches used the same covariates with the same functional form, and the same modelling assumptions. Therefore in situations where they are equivalent, CRA is clearly preferable.

All of these previous studies 8, 9, 10, 11 considered only individual‐level analysis and estimated odds ratio (OR) as a measure of intervention effect. The risk difference (RD) or risk ratio (RR) may be of interest as measures of intervention effect and have a number of advantages over OR 14. For example, they are arguably easier to understand, and they are ‘collapsible’, that is, the population marginal and conditional (on covariates or cluster effects or both) values are identical. Cluster‐level analysis methods can be used to analyse CRTs where RD or RR is estimated as a measure of intervention effect 6, and these analyses can also incorporate adjustment for baseline covariates. These methods have the advantage of being simple to apply compared with the individual‐level analysis methods. To date, the performance of cluster‐level analysis approaches with incompletely observed binary outcomes has not been investigated.

The aim of this paper is twofold. The first is to investigate the validity of estimating RD and RR as measures of intervention effect using unadjusted and adjusted cluster‐level analysis methods when binary outcomes are missing under a CDM mechanism. The second is to investigate the validity of individual‐level analysis approaches considering the limitations of previous studies 8, 9, 10, 11, which we describe in Section 3. CRA and MI are used to handle the missing outcomes.

This paper is organised as follows. We begin in Section 2 by giving a brief review of the approaches to the analysis of binary outcome in CRTs with full data. Section 3 describes methods of handling missing data in CRTs. In Section 4, we investigate the validity of CRA of CRTs under CDM assumption for missing binary outcomes. In Section 5, we report the results of a simulation study to investigate the performance of our considered methods. Section 6 presents an example of application of our results to an actual CRT. We conclude in Section 7 with some discussion.

## 2 Analysis of CRTs with full data

We begin by describing the two broad approaches to the analysis of CRTs in the absence of missing data. These two approaches are cluster‐level analysis and individual‐level analysis. Let *Y*_*ijl*_ be a binary outcome of interest for the *l*th (*l* = 1, 2, …, *m*_*ij*_) individual in the *j*th (*j* = 1, 2, …, *k*_*i*_) cluster of the *i*th (*i*=0,1) intervention group, where *i*=0 corresponds to control group and *i*=1 corresponds to intervention group. For convenience, we assume that both control and intervention groups have the same number of clusters (*k*_*i*_=*k*) and constant cluster size across the groups (*m*_*ij*_=*m*). Also let *X*_*ijl*_ be an individual‐level baseline covariate value for *l*th individual in the (*i* *j*)th cluster. Note that these methods can be extended to the case of multiple baseline covariates, some of which are individual level and some are cluster level.

In the case of a continuous outcome, it is common to assume that the expectation of the outcome is linearly dependent on the covariate and intervention indicator. However, this assumption is not very plausible in the case of a binary outcome. Two commonly used alternatives in the case of binary outcome are to assume a log or logit link between the mean of the outcome and the linear predictor.

In the case of a log link, each binary *Y*_*ijl*_ is assumed to be generated by

(1) $$\pi_{ijl}=\exp(\beta_{0}+\beta_{1}i+f_{i}(X_{ijl})+\delta_{ij}),$$

where *β*_0_ is a constant, *β*_1_ is the true intervention effect, *f*_*i*_(*X*_*ijl*_) is a function of baseline covariate *X*_*ijl*_ in the *i*th intervention group, *δ*_*ij*_ is the (*i* *j*)th cluster effect with mean 0 and $\pi_{ijl}=P\left(Y_{ijl}=1|\delta_{ij},X_{ijl}\right)$. On the other hand, assuming a logit link for the true data generating model, we have

(2) $$\pi_{ijl}=\text{expit}\left(\beta_{0}+\beta_{1}i+f_{i}(X_{ijl})+\delta_{ij}\right),$$

where expit(t)=exp(t)/(1+exp(t)).

### 2.1 Cluster‐level analysis

This approach is conceptually very simple and can be explained as a two‐stage process. Two different ways of doing cluster‐level analysis are unadjusted cluster‐level analysis and (baseline covariate) adjusted cluster‐level analysis. For binary outcomes, RD or RR is usually estimated as a measure of intervention effect in cluster‐level analysis 6.

#### 2.1.1 Unadjusted cluster‐level analysis (CL_U_)

In the first stage of analysis, a relevant summary measure of outcomes is obtained for each cluster. For binary outcomes, the cluster‐level proportion of success is usually used as the summary measure for each cluster. Let *p*_*ij*_ be the observed proportion of success in the (*i* *j*)th cluster. Then RD is estimated as

$${\hat{\text{RD}}}_{\text{unadj}}={\bar{p}}_{1}-{\bar{p}}_{0},$$

where ${\bar{p}}_{i}$ is the mean of the cluster‐specific proportions of success in the *i*th intervention group. In the second stage, a test of the hypothesis RD=0 is performed using an appropriate statistical method. The most popular one is the standard *t*‐test for two independent samples with degrees of freedom (DF) 2*k*−2. The reason for using this test is that the cluster‐specific summary measures are statistically independent, which is a consequence of the clusters being independent of each other.

On the basis of the first stage cluster‐level summary measures, RR is estimated as

$${\hat{\text{RR}}}_{\text{unadj}}=\frac{{\bar{p}}_{1}}{{\bar{p}}_{0}}.$$

Then, in the second stage, a test of the hypothesis log(RR)=0 is performed using *t*‐test with DF 2*k*−2, where $\hat{\text{V}}\left(log({\hat{\text{RR}}}_{\text{unadj}})\right)$ can be calculated as 6

$$\hat{\text{V}}\left(log({\hat{\text{RR}}}_{\text{unadj}})\right)\approx \frac{s_{0}^{2}}{k{\bar{p}}_{0}^{2}}+\frac{s_{1}^{2}}{k{\bar{p}}_{1}^{2}}\;\text{with}\;s_{i}^{2}=\frac{\overset{k}{\underset{j=1}{\sum }}{\left(p_{ij}-{\bar{p}}_{i}\right)}^{2}}{k-1}.$$

It can be shown that, with full data, ${\hat{\text{RD}}}_{\text{unadj}}$ is unbiased for RD, and ${\hat{\text{RR}}}_{\text{unadj}}$ is consistent (and, therefore, asymptotically unbiased) for RR as k→∞ (see Appendix A in the Supporting Information).

#### 2.1.2 Adjusted cluster‐level analysis (CL_A_)

In CRTs, baseline covariates that may be related to the outcome of interest are often collected and incorporated into the analysis. The main purpose of adjusting for covariates is to increase the credibility of the trial findings by demonstrating that any observed intervention effect is not attributable to the possible imbalance between the intervention groups in terms of baseline covariates 15.

In an adjusted cluster‐level analysis, an individual‐level regression analysis of the outcome of interest is carried out at the first stage of analysis ignoring the clustering of the data, which incorporates all covariates into the regression model except intervention indicator 6, 16. A standard logistic regression model is usually fitted for binary outcomes, which assumes that

(3) $$\text{logit}\left(\pi_{ijl}\right)=log\left(\frac{\pi_{ijl}}{1-\pi_{ijl}}\right)=\lambda_{1}+\lambda_{2}X_{ijl}.$$

Let *N*_*ij*_ and ${\hat{N}}_{ij}$ be the observed and predicted number of successes in the (*i* *j*)th cluster, respectively. After fitting model (3), ${\hat{N}}_{ij}$ is calculated as

$${\hat{N}}_{ij}=\overset{m}{\underset{l=1}{\sum }}{\hat{\pi }}_{ijl}=\overset{m}{\underset{l=1}{\sum }}\text{expit}\left({\hat{\lambda }}_{1}+{\hat{\lambda }}_{2}X_{ijl}\right).$$

Then the observed and predicted numbers of success are compared by computing a residual for each cluster. In the case of no intervention effect, the residuals should be similar on average in the two intervention groups.

If we want to estimate the adjusted RD, the residual, known as difference residual, for each cluster is calculated as $\epsilon_{ij}^{d}=(N_{ij}-{\hat{N}}_{ij})/m$, where the *d* superscript refers to difference residual. The adjusted RD is then estimated as

$${\hat{\text{RD}}}_{\text{adj}}={\bar{\epsilon }}_{1}^{\;d}-{\bar{\epsilon }}_{0}^{\;d},$$

where ${\bar{\epsilon }}_{i}^{d}$ is the mean of the difference residuals across the clusters of the *i*th intervention group and where ${\hat{\text{RD}}}_{\text{adj}}$ can be rewritten as

(4) $${\hat{\text{RD}}}_{\text{adj}}={\hat{\text{RD}}}_{\text{unadj}}+\frac{1}{mk}\overset{k}{\underset{j=1}{\sum }}\left({\hat{N}}_{0j}-{\hat{N}}_{1j}\right).$$

Because the distribution of *X* (in expectation) is the same between the intervention groups as a consequence of randomisation, and the prediction from the first‐stage regression model (3) depends only on $X_{ijl},\text{E}\left({\hat{N}}_{0j}\right)=\text{E}\left({\hat{N}}_{1j}\right)$. Hence, from (4), ${\hat{\text{RD}}}_{\text{adj}}$ is unbiased for RD because ${\hat{\text{RD}}}_{\text{unadj}}$ is unbiased for RD. In the second stage, a test of hypothesis RD_adj_=0 is performed using *t*‐test with DF 2*k*−2.

If we want to estimate the adjusted RR, the residual, also known as ratio residual, for each cluster is calculated as $\epsilon_{ij}^{r}=N_{ij}/{\hat{N}}_{ij}$, where the *r* superscript refers to ratio residual. The adjusted RR is then estimated as

(5) $${\hat{\text{RR}}}_{\text{adj}}=\frac{{\bar{\epsilon }}_{1}^{\;r}}{{\bar{\epsilon }}_{0}^{\;r}},$$

where ${\bar{\epsilon }}_{i}^{\;r}$ is the mean of the ratio residuals across the clusters of the *i*th intervention group. It can be shown that, with full data, ${\hat{\text{RR}}}_{\text{adj}}$ is consistent and, therefore, asymptotically unbiased (as k→∞) for true RR if (i) the true data generating model is a log link model; (ii) the functional form of the covariates is the same between the intervention groups; and (iii) the distribution of random effect is the same between the intervention groups (see Appendix B in the Supporting Information for details). In the second stage, a test of hypothesis $log\left({\text{RR}}_{\text{adj}}\right)=0$ is performed using *t*‐test with DF 2*k*−2, where $\hat{\text{V}}\left(log({\hat{\text{RR}}}_{\text{adj}})\right)$ can be calculated as

$$\hat{\text{V}}\left(log({\hat{\text{RR}}}_{\text{adj}})\right)\approx \frac{s_{\epsilon 0}^{2}}{k{\left({\bar{\epsilon }}_{0}^{r}\right)}^{2}}+\frac{s_{\epsilon 1}^{2}}{k{\left({\bar{\epsilon }}_{1}^{r}\right)}^{2}}\;\text{with}\;s_{\epsilon i}^{2}=\frac{\overset{k}{\underset{j=1}{\sum }}{\left(\epsilon_{ij}^{r}-{\bar{\epsilon }}_{i}^{r}\right)}^{2}}{k-1}.$$

### 2.2 Individual‐level analysis

In individual‐level analysis, a regression model is fitted to the individual‐level outcome that allows us to analyse the effects of intervention and other covariates in the same model. For binary outcomes, two commonly used individual‐level analysis methods are random effects logistic regression (RELR), which estimates cluster‐specific (also known as conditional) intervention effects, and generalised estimation equations (GEEs), which estimate population‐averaged (also known as marginal) intervention effects. Both of these approaches are extensions of the standard logistic regression models modified to allow for correlation between the outcomes of individuals in the same cluster. We also note that for both methods, one can obtain estimates of RD or RR by integrating over the fixed and random effects in the case of RELR and by integrating over the fixed effects in the case of GEE.

#### 2.2.1 Random effects logistic regression

RELR models take into account between‐cluster variability by incorporating cluster‐specific random effects, which are almost always assumed to be normally distributed, into the logistic regression. These models are fitted by maximising the likelihood function numerically, because the likelihood function and its derivative cannot be derived analytically as this involves an integral over the distribution of the random effects. Numerical integration methods are used to approximate the integral and so approximate the likelihood function. It is recommended to have at least 15 clusters in each intervention group to acquire the correct size and coverage for significance tests and confidence interval 6. Li and Redden 17 examined the performance of five denominator degrees of freedom (DDF) approximations, namely, residual DDF, containment DDF, between‐within DDF, Satterthwaite DDF and Kenward–Roger DDF. They recommended to use between‐within DDF approximation, which is equal to the total number of clusters in the study minus the rank of the design matrix, as it gave type I error rate close to nominal level and higher power compared with the other four methods. Ukoumunne *et al.* 18 examined the properties of *t*‐based confidence intervals for log(OR) from CRTs using DF 2*k*−2 assuming the same number of clusters in the two intervention groups. They found that the coverage rates were close to the nominal level, although this approach gave overcoverage with very small ICC (0.001). In this paper, we used the quantiles from *t*‐distribution with DF 2*k*−2 rather than quantiles from N(0,1) to construct the confidence interval for intervention effect.

#### 2.2.2 Generalised estimating equations

GEEs are commonly used as a method for analysing binary outcomes in CRTs while taking into account the correlation among the outcomes of the same cluster using a working correlation matrix. In CRTs, it is usual to assume that the correlation matrix is exchangeable, because outcomes on individuals in different clusters are uncorrelated, while outcomes on individuals in the same cluster are equally correlated.

In GEE, the sandwich standard error (SE) estimator is typically used to estimate the SE of the parameter estimates. Although the sandwich SE estimator is consistent even when the working correlation structure is specified incorrectly, the sandwich SE of the regression coefficient tends to be biased downwards when the number of clusters in each intervention group is small 6, 18. Moreover, the estimate of SE is highly variable when the number of clusters is small. It is recommended to have at least 40 clusters in the study to acquire reliable SE estimates 5. A number of methods have been proposed for dealing with the limitations of the sandwich variance estimator 18, 19. In this paper, we used the method proposed by Ukoumunne (2007) 18 to correct the bias for small number of clusters in each intervention group. Firstly, the downward bias of the sandwich SE estimator was adjusted by multiplying it by $\sqrt{k/(k-1)}$, where *k* is the number of clusters in each intervention group. Secondly, the increased small sample variability of the sandwich SE estimator was accounted for by constructing the confidence interval for intervention effect on the basis of the quantiles from a *t*‐distribution with DF 2*k*−2 rather than quantiles from N(0,1). However, if some baseline covariates were cluster level, the DF would be adjusted downwards as 2*k*−2−*q* to account for this, where *q* is the number of parameters corresponding to the cluster‐level baseline covariates.

## 3 Methods of handling missing data in CRTs

Common methods for handling missing data in CRTs are CRA, single imputation and MI. In this paper, we focused on CRA and MI because they are the most commonly used methods for handling missing data. All the analysis methods described in the previous section can be implemented using either complete records or MI. This section briefly describes these two approaches.

### 3.1 Complete records analysis

In CRA, often referred to as complete case analysis, only individuals with complete data on all variables in the analysis are considered. It has the advantage of being simple to apply and is usually the default method in most statistical packages. It is well known that CRA is valid if data are MCAR. CRA is also valid if, conditioning on covariates, missingness is independent of outcome and and the outcome model being fitted is correctly specified 20. On the basis of simulations for CDM in CRTs, Ma *et al.* 9, 10 showed that GEE using CRA performs well in terms of bias when the percentage of missing outcomes is low. In contrast, they concluded that RELR using CRA does not perform well. This is because they generated the data in such a way that they knew what the true population‐averaged log(OR) was, but after fitting RELR, they compared estimates of conditional (on cluster random effects and covariates) log(OR) with the true population‐averaged log(OR). In addition, in the data generating mechanism used in these studies 9, 10, the baseline covariate was generated independently of the outcome, which in general is not a plausible assumption. It is therefore difficult to draw conclusions about what would happen in CRTs where the baseline covariates are related to the outcome. Caille *et al.* 11 reported through simulations that GEE using unadjusted CRA and using adjusted (for covariates) CRA are biased for estimating intervention effects. However, in their simulation study, individual‐level continuous outcomes were generated at first using a linear mixed model that includes intervention indicator and a cluster random effect for each cluster, but without covariates. Each continuous outcome was then dichotomised to obtain a binary outcome. Then, baseline covariates were generated dependent on the continuous outcomes. So it appears the data generation mechanism used would mean that baseline covariates were associated with intervention group, which is not possible (in expectation) because of randomisation. In addition, as the authors noted, they compared estimates of covariate conditional ORs with the true unconditional ORs, which would be expected to differ even with full data because of non‐collapsibility. It is therefore difficult to draw general conclusions from their results about the methods' performance in CRTs.

### 3.2 Multiple imputation

In MI, a sequence of *Q* imputed datasets are obtained by replacing each missing outcome by a set of *Q*⩾2 imputed values that are simulated from an appropriate distribution or model. Imputing multiple times allows the uncertainty associated with the imputed values because the imputed values are sampled draws for the missing outcomes instead of the actual values. This uncertainty is taken into account by adding between‐imputation variance to the average within‐imputation variance. Each of the *Q* imputed datasets are analysed as a full dataset using standard methods, and the results are then combined using Rubin's rules 21. One important feature of MI is that the imputation model and the analysis model do not have to be the same. However, in order for Rubin's rules to be valid, the imputation model needs to be compatible or congenial with the analysis model 22.

There are at least four different types of MI that have been used in CRTs 7. These are *standard* MI, also known as *single‐level* MI, that ignores clustering in the imputation model, *fixed effects* MI that includes a fixed effect for each cluster in the imputation model, *random effects* MI where clustering is taken into account through a random effect for each cluster in the imputation model and *within‐cluster* MI where standard MI is applied within each cluster. From now, we refer to random effects MI as multilevel multiple imputation (MMI).

The MI inference is usually based on a *t*‐distribution with DF given by

$$\upsilon =(Q-1){\left(1+\frac{Q}{Q+1}\frac{W}{B}\right)}^{2},$$

where *B* and *W* are the between‐imputation variance and the average within‐imputation variance, respectively. This DF is derived under the assumption that the complete data (full data) DF, *υ*_com_, is infinite 23. In CRTs, the value of *υ*_com_ is calculated on the basis of the number of clusters in the study rather than the number of individuals and, therefore, is usually small. In CRTs with equal number of clusters in each intervention group, *υ*_com_ is calculated as 2*k*−2 24. If *υ*_com_ is small and there is a modest proportion of missing data, the value of *υ* can be much higher than *υ*_com_, which is not appropriate 23. In such a situation, a more appropriate DF, proposed by Barnard and Rubin (1999) 23, is calculated as

$$\nu_{\text{adj}}={\left(\upsilon^{-1}+{\hat{\upsilon }}_{\text{obs}}^{-1}\right)}^{-1}⩽\nu_{\text{com}}\;\;\text{where}\;\;{\hat{\nu }}_{\text{obs}}=\left(\frac{\nu_{\text{com}}+1}{\nu_{\text{com}}+3}\right)\nu_{\text{com}}{\left(1+\frac{Q+1}{Q}\frac{B}{W}\right)}^{-1}.$$

Ma *et al.* 8 examined within‐cluster MI, fixed effects MI and MMI for missing binary outcomes under CDM mechanism in CRTs. They showed that all these strategies give quite similar results for low percentages of missing data or for small value of ICC. With high percentage of missing data, the within‐cluster MI underestimates the variance of the intervention effect that may result in inflated type I error rate. In two subsequent studies, Ma *et al.* 9, 10 compared the performance of GEE and RELR with missing binary outcomes using standard MI and within‐cluster MI. Results showed that GEE performs well when using standard MI and the variance inflation factor is less than 3 and using within‐cluster MI when variance inflation factor is ⩾3 and cluster size is at least 50. Ma *et al.* 10 concluded that RELR does not perform well using either standard MI or within‐cluster MI. However, in the latter two studies 9, 10, as we described in Section 3.1, they compared estimates of conditional (on cluster random effects and covariates) log(OR) with the true population‐averaged log(OR), and their data generation mechanisms do not correspond to how data arise in CRTs. In the first study 8, the simulation was based on a real dataset, so the conclusions to other design settings may be limited. It is therefore again difficult to draw conclusions from their results about the performance of GEE and RELR with different MI strategies under CDM mechanism. Caille *et al.* 11 compared different MI strategies through a simulation study for handing missing binary outcomes in CRTs assuming CDM, assessing bias, SE and coverage rate of the estimated intervention effect. They showed that MMI with RELR and single‐level MI with standard logistic regression give better inference for intervention effect compared with CRA in terms of bias, efficiency and coverage. However, as we described in Section 3.1, their data generation mechanism does not correspond to how data arise in CRTs. It is therefore again difficult to draw general conclusions from their results about the MI strategies' performance in CRTs.

In the case of missing continuous outcome in CRTs, Andridge 24 showed that the true MI variance of group means are underestimated by single‐level MI and are overestimated by fixed effects MI. She also showed that MMI is the best among these three methods and recommended its use for practitioners. DiazOrdaz *et al.* 25 showed that for bivariate outcomes, MMI gives coverage rate close to nominal level, whereas single‐level MI gives low coverage and fixed effects MI gives overcoverage. In this paper, we therefore used MMI for missing binary outcome.

## 4 Validity of CRA of CRTs

In this section, we investigate the validity of CL_U_,CL_A_, RELR and GEE using complete records, when binary outcomes are missing under CDM.

In settings where the expectation of the outcome is assumed to be linearly dependent on the covariate and intervention indicator, both unadjusted and adjusted cluster‐level analyses using complete records for estimating mean difference as a measure of intervention effect are unbiased in general only when the two intervention groups have the same CDM mechanism and the same covariate effect on the outcome 13. However, as described in Section 2, the assumption of the expectation of the outcome being linear in baseline covariate and intervention indicator is not very plausible in the case of a binary outcome. Two common alternatives are to use a log or logit link between the mean of the outcome and the linear predictor.

Define a missing outcome data indicator *R*_*ijl*_ such that *R*_*ijl*_=1 if *Y*_*ijl*_ is observed and *R*_*ijl*_=0 if *Y*_*ijl*_ is missing. Then $\sum_{l=1}^{m}R_{ijl}$ is the number of complete records in the (*i* *j*)th cluster.

### 4.1 Cluster‐level analyses for estimating RD

In unadjusted cluster‐level analysis using complete records, RD is estimated as

$${\hat{\text{RD}}}_{\text{unadj}}^{\text{cr}}={\bar{p}}_{1}^{\text{cr}}-{\bar{p}}_{0}^{\text{cr}},$$

where ${\bar{p}}_{i}^{\text{cr}}$ is the mean of the cluster‐specific proportions of success, calculated using complete records, in the *i*th intervention group. The superscript **cr** refers to complete records.

In adjusted cluster‐level analysis, recall that a logistic regression model is fitted to the data at the first stage of analysis ignoring intervention and clustering of the data. Then the observed and predicted number of successes in each cluster are compared by computing a residual for each cluster. The adjusted RD using complete records is estimated as

$${\hat{\text{RD}}}_{\text{adj}}^{\text{cr}}={\bar{\epsilon }}_{1}^{\;d\text{(cr)}}-{\bar{\epsilon }}_{0}^{\;d\text{(cr)}},$$

where ${\bar{\epsilon }}_{i}^{\;d\text{(cr)}}$ is the average of the cluster‐specific difference residuals in the *i*th intervention group using complete records. Then ${\hat{\text{RD}}}_{\text{adj}}^{\text{cr}}$ can be written in terms of ${\hat{\text{RD}}}_{\text{unadj}}^{\text{cr}}$ as

(6) $$\begin{array}{cc} {\hat{\text{RD}}}_{\text{adj}}^{\text{cr}} & ={\hat{\text{RD}}}_{\text{unadj}}^{\text{cr}}+\frac{1}{k}\overset{k}{\underset{j=1}{\sum }}\left[\frac{1}{\overset{m}{\underset{l=1}{\sum }}R_{ijl}}\left({\hat{N}}_{0j}^{\text{cr}}-{\hat{N}}_{1j}^{\text{cr}}\right)\right], \end{array}$$

where ${\hat{N}}_{ij}^{\text{cr}}$ is the predicted number of successes using complete records in the (*i* *j*)th cluster.

We aim to derive conditions under which the cluster‐level analyses for RD using complete records are unbiased. To this end, we write the individual‐level probabilities of success, *π*_*ijl*_, as

$$\pi_{ijl}=\pi_{i}+g_{i}\left(X_{ijl},\delta_{ij}\right),$$

where $g_{i}\left(X_{ijl},\delta_{ij}\right)$ is a function of baseline covariate *X*_*ijl*_ and random cluster effect *δ*_*ij*_ and which determines how individual‐level probabilities of success differ from group‐level probability of success in each intervention group. Then it can be shown that ${\hat{\text{RD}}}_{\text{unadj}}^{\text{cr}}$ will be unbiased for true RD if and only if

(7) $$\text{E}\left(g_{1}\left(X_{1jl},\delta_{1j}\right)|R_{1jl}=1\right)=\text{E}\left(g_{0}\left(X_{0jl},\delta_{0j}\right)|R_{0jl}=1\right),$$

(see Appendix C of the Supporting Information for more details). Assuming the data are generated from log link model (1) or logit link model (2) and there is an intervention effect (*β*_1_≠0) in truth, the condition (7) is not satisfied even if the two intervention groups have the same missingness mechanism and the same covariate effects in the data generating model for the outcome. Hence, ${\hat{\text{RD}}}_{\text{unadj}}^{\text{cr}}$ is biased for true RD (≠0) when the true data generating model has log link or logit link. However, under the null hypothesis of no intervention effect (*β*_1_=0), if the two intervention groups have the same covariate effects and the same missingness mechanism, the condition (7) is satisfied, and hence, ${\hat{\text{RD}}}_{\text{unadj}}^{\text{cr}}$ is unbiased for true RD=0.

Referring to equation (6), if the two intervention groups have the same missingness mechanism and the same covariate effect, then $\text{E}\left({\hat{N}}_{0j}^{\text{cr}}\right)=\text{E}\left({\hat{N}}_{1j}^{\text{cr}}\right)$. Hence, with *β*_1_≠0, from equation (6), we can conclude that because ${\hat{\text{RD}}}_{\text{unadj}}^{\text{cr}}$ is biased for RD (≠0) with both log and logit links for the true data generating model, ${\hat{\text{RD}}}_{\text{adj}}^{\text{cr}}$ is also biased for RD (≠0) with both log and logit links in the true data generating model. However, with *β*_1_=0, since ${\hat{\text{RD}}}_{\text{unadj}}^{\text{cr}}$ is unbiased for RD=0 with both log and logit links, when the two intervention groups have the same missingness mechanism and the same covariate effect, ${\hat{\text{RD}}}_{\text{adj}}^{\text{cr}}$ is also unbiased for RD=0 under the same conditions. It can also be shown that the expectation of $g_{i}\left(X_{ijl},\delta_{ij}\right)$ over (*j*,*l*) is zero for *i*∈{0,1} for both log and logit links in the data generating model, and hence, both ${\hat{\text{RD}}}_{\text{unadj}}$ and ${\hat{\text{RD}}}_{\text{adj}}$ are unbiased for true RD with full data.

### 4.2 Cluster‐level analyses for estimating RR

In both unadjusted and adjusted cluster‐level analyses, RR is estimated using complete records as, respectively,

(8) $${\hat{\text{RR}}}_{\text{unadj}}^{\text{cr}}=\frac{{\bar{p}}_{1}^{\text{cr}}}{{\bar{p}}_{0}^{\text{cr}}}\;\;\text{and}\;\;{\hat{\text{RR}}}_{\text{adj}}^{\text{cr}}=\frac{{\bar{\epsilon }}_{1}^{\;r\text{(cr)}}}{{\bar{\epsilon }}_{0}^{\;r\text{(cr)}}},$$

where ${\bar{\epsilon }}_{i}^{\;r\text{(cr)}}$ is the average of the ratio residuals in the *i*th intervention group using complete records.

We aim to establish conditions under which the cluster‐level analyses for RR using complete records are consistent. To this end, we write *π*_*ijl*_ as

$$\pi_{ijl}=\pi_{i}\;h_{i}\left(X_{ijl},\delta_{ij}\right),$$

where $h_{i}\left(X_{ijl},\delta_{ij}\right)$ is a function of baseline covariate *X*_*ijl*_ and random cluster effect *δ*_*ij*_ and which determines how individual‐level probabilities of success differ from group‐level probability of success. Then it can be shown that ${\hat{\text{RR}}}_{\text{unadj}}^{\text{cr}}$ will be consistent for true RR if only if

(9) $$\frac{\text{E}\left(h_{1}\left(X_{1jl},\delta_{1j}\right)|R_{1jl}=1\right)}{\text{E}\left(h_{0}\left(X_{0jl},\delta_{0j}\right)|R_{0jl}=1\right)}=1,$$

(see Appendix D of the Supporting Information for more details). Assuming the data are generated from log link model (1), the condition (9) is satisfied if the two intervention groups have the same missingness mechanism and the same covariate effects, and hence, ${\hat{\text{RR}}}_{\text{unadj}}^{\text{cr}}$ is consistent (and, therefore, asymptotically unbiased) for true RR.

On the other hand, assuming the data are generated from logit link model (2) with *β*_1_≠0, the condition (9) is not satisfied even if the two intervention groups have the same missingness mechanism and the same covariate effects. Hence, ${\hat{\text{RR}}}_{\text{unadj}}^{\text{cr}}$ is not consistent for true RR (≠1). However, under the null hypothesis of no intervention effect (*β*_1_=0), if the two intervention group have the same missingness mechanism and the same covariate effect, the condition (9) is satisfied, and hence, ${\hat{\text{RR}}}_{\text{unadj}}^{\text{cr}}$ is consistent for true RR=1.

In Appendix E of the Supporting Information, we show that ${\hat{\text{RR}}}_{\text{adj}}^{\text{cr}}$ is consistent and, therefore, asymptotically unbiased (as k→∞) for true RR if (i) the true data generating model is a log link model, (ii) the functional form of the covariates in the outcome model is the same between the intervention groups, (iii) the missingness mechanism is the same between the intervention groups and (iv) the distribution of random effects is the same between the intervention groups. If the data are generated from logit link model (2) with $\beta_{1}\neq 0,{\hat{\text{RR}}}_{\text{adj}}^{\text{cr}}$ is not consistent for true RR (≠1). However, under the null hypothesis of no intervention effect $(\beta_{1}=0),{\hat{\text{RR}}}_{\text{adj}}^{\text{cr}}$ is consistent (as k→∞) for true RR (=1) if (i) the true data generating model is a logit link model, (ii) the functional form of the covariates is the same between the intervention groups, (iii) the missingness mechanism is the same between the intervention groups and (iv) the distribution of random effects is the same between the intervention groups.

### 4.3 RELR and GEE using complete records

For individually randomised trials, it is well known that likelihood‐based CRA is valid under MAR, if missingness is only in the outcome and all predictors of missingness are included in the model as covariates 20. So it is anticipated that RELR using CRA will give consistent estimates of intervention effect, if the covariate *X*, which is associated with the missingness, is included in the model and the model is correctly specified. We also expect that GEE using CRA adjusted for covariate *X* that is associated with the missingness in outcomes will give consistent estimates of intervention effect.

When it is assumed that the two intervention groups have the same covariate effects on outcome, we fit RELR with fixed effects of intervention indicator and covariate and a random effect for cluster, while we fit GEE with intervention indicator and covariate assuming exchangeable correlation for the outcomes of the same cluster. If it is assumed that the baseline covariate effect on outcome could be different in the two intervention groups, an interaction between intervention and covariate must be included in the model. This implies that the intervention effect varies with level of covariate values. In those scenarios where an interaction is present, we will target the intervention effect at the mean value of the covariate. Let *X* ^∗^ denote the empirically centred covariate $X-\bar{X}$, where $\bar{X}$ is the mean of *X* using data from all individuals. Then, we fit RELR with fixed effects of intervention indicator, *X* ^∗^ and their interaction, and a random effect for cluster, while we fit GEE including the intervention indicator, *X* ^∗^ and their interaction, and assuming an exchangeable correlation for the outcomes of the same cluster. One may need to account for the centring step in the variance estimation. We will investigate in the simulation whether ignoring this has any negative impact on confidence interval coverage.

## 5 Simulation study

A simulation study was conducted to assess the performance of CL_U_,CL_A_, RELR and GEE under CDM mechanism. CRA and MMI were used to handle the missing data. The average estimate of intervention effect, its average estimated SE and coverage rates were calculated for each of the methods and compared with each other. We considered balanced CRTs, where the two intervention groups have the same number of clusters and constant cluster size (before missing outcomes were introduced), and a single continuous individual‐level baseline covariate.

### 5.1 Data generation

Data were generated using the model in equation (2) with a logit link, as described in Section 2, with *f*_*i*_(*X*_*ijl*_)=*β*_2(*i*)_ *X*_*ijl*_, where *β*_2(*i*)_ is the effect of covariate of *X* in the *i*th intervention group. For each individual in the study, a value of *X*_*ijl*_ was generated using the model

$$X_{ijl}=\alpha_{ij}+u_{ijl},$$

where *α*_*ij*_ is the (*i* *j*)th cluster effect on *X* and *u*_*ijl*_ is the individual‐level error on *X*. We assumed that $\alpha_{ij}∼N\left(\mu_{x},\sigma_{\alpha }^{2}\right)$ independently of $u_{ijl}∼N\left(0,\sigma_{u}^{2}\right)$, where *μ*_*x*_ is the mean of $X,\sigma_{\alpha }^{2}$ and $\sigma_{u}^{2}$ are the between‐cluster and within‐cluster variance of *X*, respectively. The total variance of *X* can be written as $\sigma_{x}^{2}=\sigma_{\alpha }^{2}+\sigma_{u}^{2}$, and thus, the ICC of *X* is $\rho_{x}=\sigma_{\alpha }^{2}/\sigma_{x}^{2}$. Then, we generated logit(*π*_*ijl*_) for each individual in the study using model (2) assuming $\delta_{ij}∼N\left(0,\sigma_{b}^{2}\right)$. Finally, *Y*_*ijl*_ was generated as Bernoulli random variable with parameter *π*_*ijl*_.

Once the complete datasets (full data) were generated, we introduced missing outcomes by generating a missing outcome data indicator *R*_*ijl*_ (defined in Section (4)), independently for each individual, under CDM mechanism according to a logistic regression model

(10) $$\text{logit}(R_{ijl}=0|Y_{ij},X_{ij})=\psi_{i}+\phi_{i}X_{ijl},$$

where ***Y***_*ij*_ and ***X***_*ij*_ are the vectors of outcome and covariate values, respectively, of the (*i* *j*)th cluster. The constants *ψ*_*i*_ and *ϕ*_*i*_ were chosen such that the *i*th intervention group had the desired proportion of observed outcomes. The value of *ϕ*_*i*_ in equation (10) represents the degree of association between the missingness and the covariate *X* in the *i*th intervention group. In this study, we assumed the same covariate effects for the probability of having a missing outcome in the two intervention groups and thus set *ϕ*_0_=*ϕ*_1_=1 in equation (10) corresponding to the OR of having a missing outcome of 2.72 for a 1 unit change in *X*.

We investigated four scenarios, varying whether the baseline covariate effects on outcome and the missingness mechanisms were the same in the two intervention groups. For generating *X*_*ijl*_, we chose $\mu_{x}=0,\sigma_{u}^{2}=3.37\;\text{and}\;\sigma_{\alpha }^{2}=0.18$, and thus, we had $\sigma_{x}^{2}=3.55\;\text{and}\;\rho_{x}=0.05$. Then, to generate *Y*_*ijl*_, we set $\sigma_{b}^{2}=0.20,\beta_{0}=0$ and *β*_1_=1.36 and varied *β*_2(0)_ and *β*_2(1)_ across the four scenarios to obtain the value of success rates *π*_0_=0.50 and *π*_1_=0.70 in the control and intervention groups, respectively, on average over 1000 datasets. The value of ICC for outcome is expected to be different in the control and intervention groups because, for binary outcome, ICC depends on the success rate 26. We used the expression $\rho_{i}=\mathrm{Var}\left(\pi_{ij}\right)/\left(\pi_{i}(1-\pi_{i})\right)$ 6, 27, where *π*_*ij*_ is the true proportion of success in the (*i* *j*)th cluster, to estimate the value of ICC for the *i*th intervention group. Firstly, we estimated $\mathrm{Var}\left(\pi_{ij}\right)$ from a very big dataset with large number of clusters in each intervention group and with large cluster size. Then, with the success rates stated earlier for the control and intervention groups, the estimated ICC for outcome in the control and intervention groups were 0.037 and 0.032, respectively. We varied the number of clusters in each intervention group as *k*=(5,10,20,50) and fixed the cluster size *m*=50. In the simulation studies, the four scenarios considered were **(S1)** *β*_2(0)_=*β*_2(1)_=1 and *ψ*_0_=*ψ*_1_=−1.34; that is, both intervention groups have the same covariate effects on outcome and the same missingness mechanisms; **(S2)** *β*_2(0)_=*β*_2(1)_=1 and *ψ*_0_=−1.34,*ψ*_1_=0.65; that is, both intervention groups have the same covariate effects on outcome but different missingness mechanisms; **(S3)** *β*_2(0)_=0.588,*β*_2(1)_=1 and *ψ*_0_=*ψ*_1_=−1.34; that is, both intervention groups have different covariate effects on outcome but the same missingness mechanisms; and **(S4)** *β*_2(0)_=0.588,*β*_2(1)_=1 and *ψ*_0_=−1.34,*ψ*_1_=0.65; that is, both intervention groups have different covariate effects on outcome and different missingness mechanisms. In **S1** and **S3**, there were 30% missing outcomes in each of the two intervention groups, while in **S2** and **S4**, there were 30% missing outcomes in the control group and 60% missing outcomes in the intervention group.

### 5.2 Data analysis

Each generated full and incomplete datasets were then analysed by CL_U_,CL_A_, RELR and GEE. Missing outcomes were handled using CRA and MMI. We included the interaction between intervention and baseline covariate into the analysis models RELR and GEE in the case of **S3** and **S4**. The R packages **lme4** and **geepack** were used to fit RELR and GEE, respectively. We used MMI, with a RELR imputation model, so that the imputation model was correctly specified. For **S3** and **S4**, an interaction between intervention and baseline covariate was included in the imputation model. The R package jomo 28 was used to multiply impute each generated incomplete dataset 15 times, although this package uses probit link between the mean of the outcome and the linear predictor. Both links give similar results as long as individual‐level probabilities of success are not too small and not too large. The algorithm used by jomo 28 is essentially the same used by the REALCOM‐IMPUTE software for MMI, details of which can be found in 29. We used 100 burn‐in iterations, which through preliminary investigations, we found to be sufficient for convergence to the posterior distribution of the parameters of our imputation model, and thinning rate 25 to reduce the autocorrelation between successive draws. When fitting the GEE models using the package **geepack** in R, we encountered convergence problems (maximum of three times out of 1000 simulation runs) in the case of **S2** and **S4**. In such situation, we fitted GEE assuming independent correlation structure.

### 5.3 Simulation results

**Figure 1:**
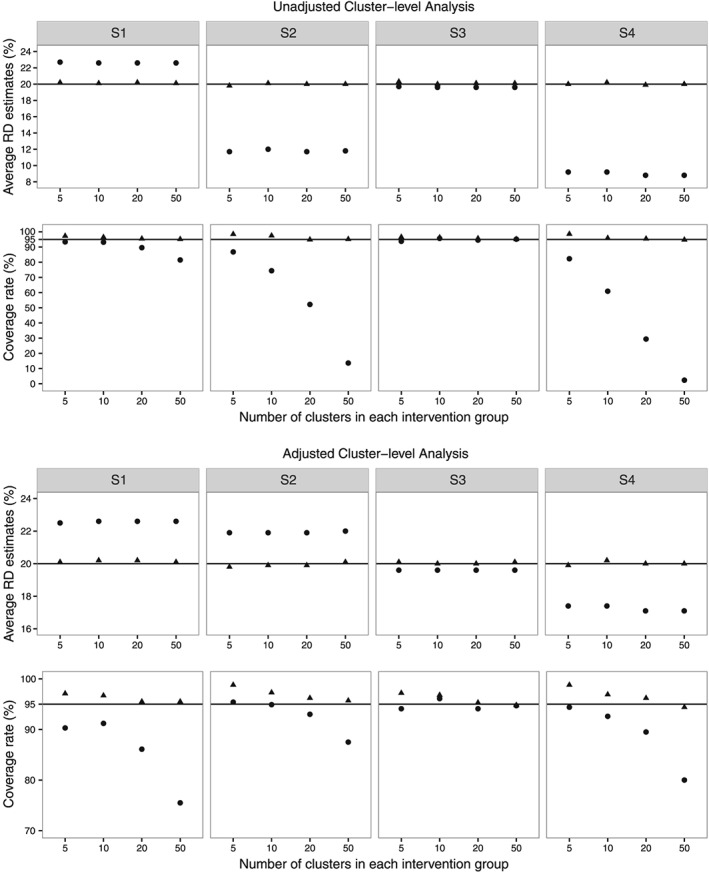
Simulation results for risk difference (RD). The columns represent the four scenarios considered in the simulation studies. The first and second rows represent the average estimates of RD and coverage rates for nominal 95% confidence interval, respectively, using unadjusted cluster‐level analysis. The third and fourth rows represent the similar estimates using adjusted cluster‐level analysis. Results are shown for complete records analysis (•) and multilevel multiple imputation ( ▴) over 1000 simulation runs. The lines (—) correspond to the true value.

Figure 1 represents the average estimates of RD and coverage rates of nominal 95% confidence intervals over 1000 simulation runs using CL_U_ and CL_A_ with CRA and MMI for each of the four scenarios. The corresponding numerical results using full data, CRA and MMI are available in Table F1 in Appendix F of the Supporting Information. The RD estimates using full data and using MMI followed by cluster‐level analyses were unbiased for each of the four scenarios. However, CRA estimates were biased using both the CL_U_ and CL_A_ for each of the four scenarios. These results support our derived analytical results for RD estimates in Section 4.1. Under scenario **S3**, the CRA estimates of RD using both the CL_U_ and CL_A_ were coincidentally close to the true value of RD. In further simulations, where the parameter values were changed, the corresponding estimates of RD, using both the CL_U_ and CL_A_, were found to be biased (see Table F2 in Appendix F in the Supporting Information). As expected, the average estimated SEs of CL_A_ are smaller than that of CL_U_, using full data, CRA and MMI. This is because the CL_A_ removes the differences between the outcome values of the two intervention groups that can be attributed to differences in the baseline covariate. MMI with adjusted DF estimates gave overcoverage for nominal 95% confidence intervals for small number of clusters in each intervention group.

**Figure 2:**
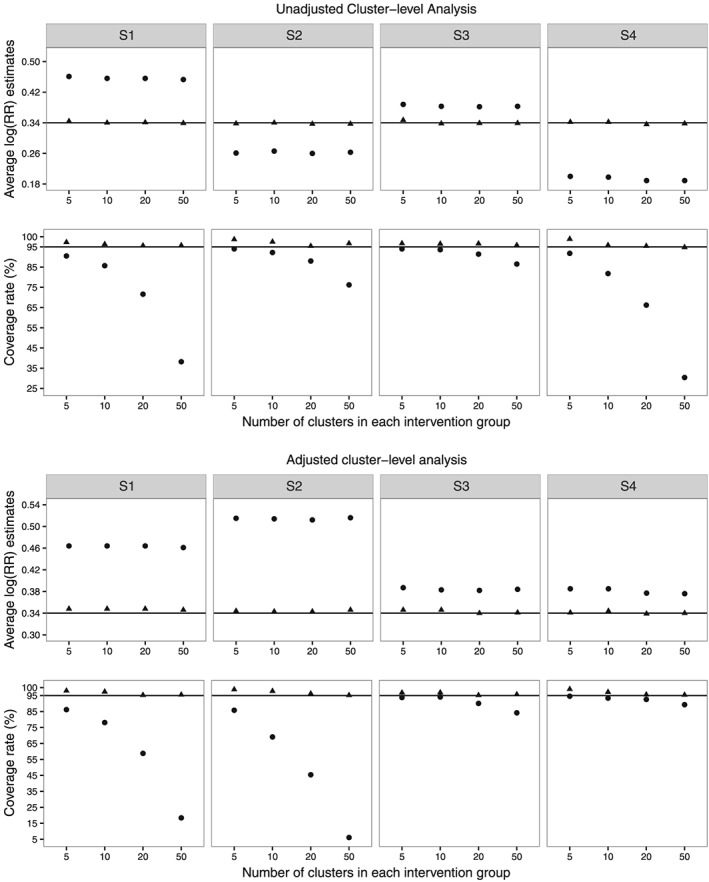
Simulation results for risk ratio (RR). The columns represent the four scenarios considered in the simulation studies. The first and second rows represent the average estimates of log(RR) and coverage rates for nominal 95% confidence interval, respectively, using unadjusted cluster‐level analysis. The third and fourth rows represent the similar estimates using adjusted cluster‐level analysis. Results are shown for complete records analysis (•) and multilevel multiple imputation ( ▴) over 1000 simulation runs. The lines (—) correspond to the true value.

Figure 2 shows the average estimates of log(RR) and coverage rates for nominal 95% confidence intervals over 1000 simulation runs using CL_U_ and CL_A_ with CRA and MMI for the all four considered scenarios. The corresponding numerical results using full data, CRA and MMI are available in Table F3 in Appendix F of the Supporting Information. The full data estimates of log(RR) using CL_U_ and CL_A_ were very close to the true value. However, our analytical result showed that CL_A_ estimates of RR are biased if the data are generated from a logit link model. In this simulation, CL_A_ estimates were close to the true value because of the parameters' configuration. In a further simulation, where the parameters' values were changed, the estimates of log(RR) using CL_A_ were found to be biased (see Table F4 in Appendix F in the Supporting Information). The MMI followed by cluster‐level analyses estimates of log(RR) were unbiased for all four considered scenarios. The CRA estimates were biased using both CL_U_ and CL_A_ for all four considered scenarios. These results support our derived analytical results for RR in Section 4.2. MMI with adjusted DF estimates resulted in the overcoverage of nominal 95% confidence intervals for small number of clusters in each intervention group.

Recall that RELR estimates cluster‐specific (also known as conditional) intervention effect, while GEE estimates population‐averaged (also known as marginal) intervention effect. In this study, the simulation data were generated using a RELR model (equation (2)), where we set *β*_1_=1.36, which can be interpreted as conditional (on cluster random effects and baseline covariate *X*) log(OR) of developing the event of interest in the intervention group compared with the control group. The corresponding marginal value of *β*_1_ will be smaller because the general effect of using a population‐averaged model over cluster‐specific model is to attenuate the regression coefficient 27. Table 1 displays the average estimates of the log(OR), their average estimated SE and coverage rates of nominal 95% confidence intervals using RELR and GEE. The full data estimates of GEE is slightly lower as expected than that of RELR. For GEE, the CRA and MMI estimates were compared with the mean of the full data estimates as the true population‐averaged log(OR) was unknown. The CRA estimates of RELR and GEE were unbiased with nominal coverage rates. This is because we were adjusting for the baseline covariate that was associated with missingness. However, RELR with MMI gave slightly upward biased (maximum 8.6%) estimates of intervention effect with small number of clusters in each intervention group, while GEE with MMI gave unbiased estimates. The study by Caille *et al.* 11 showed similar results to ours regarding good performance of GEE with respect to bias and coverage rate using MMI. The average estimated SEs of RELR estimates using CRA were lower than that of RELR using MMI, whereas the average estimated SEs of GEE estimates using CRA and MMI are fairly similar. Therefore, there is no benefit in doing MMI over CRA when the CRA and MMI use the same functional form of baseline covariates.

**Table 1:** Average estimates of log(OR), their average estimated SEs and coverage rates for nominal 95% confidence intervals over 1000 simulation runs, using RELR and GEEs with full data, CRA and MMI. Monte Carlo errors for average estimates and average estimated SEs are all less than 0.016 and 0.003, respectively. The true value of conditional log(OR) in RELR is 1.36. The true value of population‐averaged log(OR) for GEE was empirically estimated using full data.

	*k*	Average estimate						Average estimated SE						Coverage (%)					
		Full		CRA		MMI		Full		CRA		MMI		Full		CRA		MMI	
		RELR	GEE	RELR	GEE	RELR	GEE	RELR	GEE	RELR	GEE	RELR	GEE	RELR	GEE	RELR	GEE	RELR	GEE
S1	5	1.363	1.321	1.360	1.320	1.384	1.328	0.341	0.363	0.364	0.382	0.391	0.372	94.6	95.2	94.4	94.7	97.7	96.5
	10	1.365	1.321	1.368	1.323	1.392	1.329	0.252	0.258	0.268	0.271	0.284	0.272	94.6	95.2	94.4	95.1	96.1	96.0
	20	1.361	1.315	1.363	1.317	1.385	1.322	0.182	0.184	0.193	0.192	0.201	0.195	94.7	95.0	95.0	94.7	95.8	95.5
	50	1.359	1.310	1.361	1.310	1.380	1.316	0.118	0.117	0.125	0.122	0.129	0.124	94.4	95.1	94.8	95.4	94.8	95.0
S2	5	1.345	1.311	1.368	1.333	1.402	1.335	0.336	0.320	0.405	0.417	0.456	0.438	94.7	94.8	95.5	94.9	98.6	98.6
	10	1.350	1.309	1.356	1.313	1.384	1.308	0.250	0.258	0.298	0.301	0.330	0.317	93.2	94.4	94.7	95.4	97.0	97.1
	20	1.358	1.311	1.352	1.305	1.376	1.301	0.184	0.185	0.215	0.213	0.232	0.224	94.8	95.8	95.0	94.9	96.7	96.4
	50	1.366	1.316	1.367	1.318	1.389	1.316	0.118	0.117	0.138	0.135	0.146	0.141	95.3	95.7	95.0	95.0	95.8	96.0
S3	5	1.391	1.353	1.407	1.367	1.434	1.374	0.343	0.358	0.392	0.400	0.414	0.389	94.8	94.1	95.2	94.4	97.7	97.4
	10	1.352	1.307	1.359	1.314	1.385	1.320	0.254	0.259	0.284	0.286	0.299	0.285	92.8	94.1	94.0	94.5	95.4	95.0
	20	1.372	1.326	1.370	1.325	1.395	1.330	0.183	0.184	0.204	0.202	0.212	0.203	93.2	94.4	93.2	94.1	94.1	94.1
	50	1.363	1.313	1.363	1.313	1.386	1.317	0.118	0.117	0.132	0.127	0.135	0.129	95.1	95.1	94.8	95.5	95.4	95.4
S4	5	1.375	1.336	1.413	1.378	1.476	1.390	0.346	0.366	0.497	0.493	0.535	0.505	94.5	95.2	97.0	94.0	98.6	98.5
	10	1.366	1.325	1.377	1.334	1.431	1.342	0.252	0.258	0.353	0.351	0.375	0.357	94.6	95.3	95.3	94.6	96.5	96.6
	20	1.376	1.328	1.387	1.339	1.432	1.346	0.183	0.184	0.252	0.247	0.266	0.251	94.7	94.8	94.3	94.4	94.5	94.8
	50	1.360	1.312	1.362	1.313	1.397	1.317	0.118	0.117	0.160	0.156	0.167	0.157	95.4	95.7	94.8	94.5	94.4	94.2

SEs: standard errors; RELR: random effects logistic regression; GEE: generalised estimation equations; CRA: complete records analysis; MMI: multilevel multiple imputation.

## 6 Example

We now illustrate the methods compared here using the data from Health and Literacy Intervention (HALI) trial, a factorial CRT designed to investigate the impact of two interventions among school children in class 1 and class 5 on the south coast of Kenya 30. The interventions were intermittent screening and treatment (IST) for malaria on the health and education of school children in class 1 and class 5 and a literacy intervention (LIT) on education only being applied in class 1. One hundred and one government primary schools were randomised to one of the four groups receiving (i) IST alone (25 schools); (ii) LIT alone (25 schools); (iii) both IST and LIT (26 schools); or (iv) neither IST nor LIT (25 schools). On average, the number of children per school in the four groups were, respectively, 107 (standard deviation (SD) = 7.54 ), 99 (SD = 17.84), 103 (SD = 6.28) and 102 (SD = 7.51). The primary outcomes were anaemia at either 12 or 24 months and educational achievement at 9 and 24 months assessed by a battery of tests of reading, writing and arithmetic. Baseline characteristics of the school (school mean exam score and school size), the child (age, sex, sleep under net and baseline anaemia) and the household (paternal education and household size) were collected. For the purpose of illustration, we restricted attention to anaemia (binary) measured at the 24 months follow‐up. A paper published based on this study 30 showed no evidence of interaction between the two interventions in class 1 where both were implemented. We therefore merged groups (i) and (iii) where IST was implemented and considered this as the intervention group and merged groups (ii) and (iv) where IST was not implemented and considered this as the control group. The control group and the intervention group consisted of 2502 and 2674 children, respectively, and among them, 475 (18.98%) and 501 (18.74%) had missing anaemia at 24 months, respectively. The covariate baseline anaemia had some missing values as well. To illustrate our methods for the case where only outcomes are missing and all baseline covariates are fully observed, we excluded the children from the analysis with missing baseline anaemia value. Hence, in our analysis, the control group and the intervention group consisted of 2373 and 2451 children, respectively, and among them, 430 (18.12%) and 424 (17.30%) had missing anaemia at 24 months, respectively.

The original trial's prespecified analysis planned to adjust for the baseline covariates' age, sex, exam score, literacy group and baseline anaemia. In our analysis, firstly, we investigated the association of the baseline covariates (age, sex, exam score, literacy group and baseline anaemia) with anaemia at 24 months and with the probability of anaemia outcome at 24 months being missing by fitting RELR models (see Table F5 in Appendix F of the Supporting Information). Age and baseline anaemia were strongly associated with anaemia at 24 months, and there was no evidence of interaction between IST intervention and baseline covariates in the model for anaemia at 24 months. Older children were more likely to have anaemia at 24 months missing, and children receiving LIT were less likely to have anaemia at 24 months missing. There was weak evidence of interaction between IST intervention and literacy group on the missingness of anaemia at 24 months. Based on these analyses, a working assumption is that missingness of anaemia at 24 months depends mainly on age and that this dependence does not differ between the two intervention groups as there was no evidence of interaction between IST intervention and age.

We analysed the data using the methods CL_U_,CL_A_, RELR and GEE, assuming that the missingness in anaemia at 24 months depends on the baseline covariates, but conditioning on these, not on the anaemia at 24 months itself, that is, a CDM mechanism. GEE models were fitted assuming both logit and log links for the true outcome model to estimate OR and RR, respectively. The objective of fitting GEE with log link was to estimate RR using individual‐level analysis and to compare these estimates with the similar estimates obtained using cluster‐level analyses. In addition, we wanted to compare our estimates of RR using GEE with the estimates of RR reported in the original paper 30 published based on this HALI trial data. The missing anaemia data at 24 months were handled using CRA and MMI. The RELR, GEE and adjusted cluster‐level analyses were adjusted for the baseline covariates age, sex, school mean exam score, literacy group and baseline anaemia. MMI was carried out using the R package jomo 28, with an imputation model adjusted for the aforementioned baseline covariates. We used 100 imputed datasets in MMI. GEE with log link after MMI was not congenial with the imputation model, as the imputation model used probit link. The estimates and confidence intervals of RD, RR and OR obtained by CRA and MMI are displayed in Table 2. Columns *M*_0_ and *M*_1_ in Table 2 represent the number of children in the control and intervention groups, respectively. All measures showed no evidence of IST intervention effect in improving health of school children by alleviating anaemia. The CRA estimates of RD and RR using cluster‐level analyses are very similar to the corresponding estimates obtained by MMI. This is because CRA is valid in this case as there is no evidence of intervention effect and no evidence of interaction between covariates and intervention. The estimates and CIs of unadjusted and adjusted OR obtained by CRA were found to be very close to the corresponding estimates obtained by MMI. This is because, as we found in our simulation results, there is no gain in terms of bias or efficiency of the estimates using MMI over CRA as long as the same functional form of the same set of predictors of missingness are used by both methods.

**Table 2:** Risk difference, risk ratio and odds ratio estimates using CRA and MMI for the IST intervention trial data.

Analysis approach		*M*_0_	*M*_1_	Risk difference	Risk ratio	Odds ratio
				Estimate (95% CI)	Estimate (95% CI)	Estimate (95% CI)
Cluster‐level analysis[Link]						

CRA						
Unadjusted		2027	2173	0.019 (−0.040, 0.077)	1.047 (0.908, 1.208)	
Adjusted		1935	2027	0.022 (−0.033, 0.077)	1.037 (0.908, 1.185)	
MMI						
Unadjusted		2373	2451	0.021 (−0.038, 0.080)	1.053 (0.911, 1.218)	
Adjusted		2373	2451	0.017 (−0.035, 0.070)	1.040 (0.910, 1.189)	
Individual‐level analysis						

CRA						
RELR						
Unadjusted		2027	2173		—	1.090 (0.841, 1.414)
Adjusted		1935	2027		—	1.088 (0.839, 1.409)
GEE^a^						
Unadjusted		2027	2173		1.048 (0.908, 1.209)	1.082 (0.850, 1.378)
Adjusted		1935	2027		1.019 (0.911, 1.141)	1.070 (0.842, 1.359)
MMI						
RELR						
Unadjusted		2373	2451		—	1.101 (0.849, 1.428)
Adjusted		2373	2451		—	1.089 (0.841, 1.413)
GEE						
Unadjusted		2373	2451		1.053 (0.912, 1.215)	1.090 (0.856, 1.389)
Adjusted		2373	2451		1.019 (0.911, 1.140)	1.072 (0.843, 1.363)

a Cluster‐level analysis was used to estimate the risk difference and the risk ratio.

GEE was used to estimate the risk ratio using log link and to estimate the marginal odds ratio using logit link.

CRA, complete records analysis; MMI, multilevel multiple imputation; RELR, random effects logistic regression; GEE, generalised estimation equation; IST, intermittent screening and treatment; CI, confidence interval.

## 7 Discussion and conclusion

In this paper, we showed analytically and through simulations that cluster‐level analyses for estimating RD using complete records are valid only when there is no intervention effect in truth and the intervention groups have the same missingness mechanism and the same covariate effect in the outcome model. For estimating RR, cluster‐level analyses using complete records are valid if the true data generating model has log link and the intervention groups have the same missingness mechanism and the same covariate effect in the outcome model. However, if the true data generating model has logit link, cluster‐level analyses using complete records for estimating RR are valid only when there is no intervention effect in truth and the intervention groups have the same missingness mechanism and the same covariate effect in the outcome model. But, in practice, it is impossible to know in advance whether there is an intervention effect. We therefore caution researchers that cluster‐level analyses using complete records, assuming logit link for the true data generating model, in general results in biased inferences for RR in CRTs. However, when the true data generating model follows a log link and the parameter of interest is RR, cluster‐level analyses using complete records give valid inferences if the intervention groups have the same missingness mechanism and the same covariates effect in the outcome model.

In contrast, MMI followed by cluster‐level analyses gave unbiased estimates of RD and RR regardless of whether missingness mechanisms were the same or different between the intervention groups and whether there is an interaction between intervention and baseline covariate in the outcome model, provided that an interaction was allowed for in the imputation model when required. However, MMI resulted in overcoverage for the nominal 95% confidence interval with small number of clusters in each intervention group. Similar results were found for continuous outcomes in CRTs by Hossain *et al.* 13.

The full data estimates of conditional (on cluster random effects and covariates) log(OR) using RELR were unbiased with good coverage rates. These results differ from the results found by Ma *et al.* 10, where they concluded that full data estimates using RELR were biased. As noted previously, we believe this is because they generated the data in such a way that they knew what the true population‐averaged log(OR) was, but after fitting RELR, they compared the estimates of conditional log(OR) with the true population‐averaged log(OR). As noted earlier, population‐averaged log(OR) is marginal with respect to the cluster random effects 31.

The CRA estimates of conditional log(OR) using RELR were unbiased with coverage rates close to the nominal level regardless of whether the missingness mechanism is the same or different between the intervention groups and whether there is an interaction between the intervention and baseline covariate in the data generating model for outcome, provided that if there is an interaction in the data generating model for the outcome, then this interaction is included in the model fitted to the data. This conclusion contradicts the results of a previous study by Ma *et al.* 10, where they found that CRA estimates using RELR are biased under CDM assumption. Again we believe this is because they compared RELR estimates of the conditional log(OR) with the true marginal log(OR). The conclusions of Ma *et al.* 10 have subsequently been cited in a recent textbook on CRT design and analysis 27. We hope that our results and explanations help in understanding some of the surprising results and conclusion in Ma *et al.* 8, 9, 10. In our study, we also found that the RELR with MMI gave slightly upward biased estimates of conditional log(OR) for small number of clusters in each intervention groups.

The GEE using CRA and MMI gave unbiased estimates of population‐averaged log(OR) with coverage rates close to the nominal level regardless of whether the missingness mechanism was the same between the intervention groups and whether there was an interaction between the intervention group and baseline covariate in the data generating model. Similar results had been found by Ma *et al.* 10 for GEE in terms of bias, although as described earlier, in their data generating mechanism, the covariate was generated independently of the outcome.

In this study, we assumed the same covariate effects for the probability of having a missing outcome in the two intervention groups. Another possible scenario would be that the two groups have different missingness mechanism in the sense that the covariate effects on the probability of having missing outcome are different between the two intervention groups. To address this, we have carried out a further simulation with different covariate effects (*ϕ*_0_=0.5,*ϕ*_1_=1) on the probability of having a missing outcome between the two groups. The results showed, as expected by theory, that CRA gives valid estimates. This is because, CRA is valid as long as conditional on the covariates in the model, the missingness is independent of the outcome. We also assumed baseline CDM assumption for binary outcome, which is an example of MAR as our baseline covariate was fully observed. In practice, it cannot be identified on the basis of the observed data that missingness assumption is appropriate 32, 33. Therefore, sensitivity analyses should be performed [33, Ch. 10] to explore whether inferences are robust to the primary working assumption regarding the missingness mechanism. Furthermore, we focused on studies with only one individual‐level baseline covariate; the methods described can be extended to more than one baseline covariate.

In conclusion, as long as both MMI and CRA use the same covariates with the same functional form, RELR or GEE using complete records can be recommended as the primary analysis approach for CRTs with missing binary outcomes if we are willing to assume that the missingness depends on baseline covariates and conditional on these, not on the outcome. In addition, where the aim is to estimate RD or RR, MMI can be used followed by cluster‐level analysis to acquire valid estimates under the CDM assumption for missing binary outcomes, but one should be cautious when making inferences as this approach results in overcoverage for small number of clusters in each intervention group.

## Supporting information



Missing binary outcomes under covariate dependent missingness in cluster randomised trials
