# Supplementary material for: Missing binary outcomes under covariate‐dependent missingness in cluster randomised trials
Source: Stat Med. 2017 May 29;36(19):3092–109. doi: 10.1002/sim.7334 (PMC5518290; doi:10.1002/sim.7334)
Supplement: Supplementary file 1 — Missing binary outcomes under covariate dependent missingness in cluster randomised trials [file SIM-36-3092-s001.pdf]

# Missing binary outcomes under covariate dependent missingness in cluster randomised trials

Anower Hossain[1, 2], Karla DiazOrdaz[1], and Jonathan W. Bartlett [3]

[1]Department of Medical Statistics, London School of Hygiene and Tropical Medicine, London, UK.
[2]Institute of Statistical Research and Training (ISRT), University of Dhaka, Dhaka, Bangladesh.
[3]Statistical Innovation Group, AstraZeneca.

## Appendix A

In this appendix, we show that, with full data, $\widehat{\mathrm{RD}}_{\mathrm{unadj}}$ is unbiased for true RD, and $\widehat{\mathrm{RR}}_{\mathrm{unadj}}$ is consistent (and, therefore, asymptotically unbiased) for true RR. We have

$$\mathrm{E}\left(\bar{p}_i\right) = \mathrm{E}\left(\frac{1}{k}\sum_{i=1}^{k} p_{ij}\right) = \frac{1}{mk}\sum_{j=1}^{k}\sum_{l=1}^{m} \mathrm{E}\left(Y_{ijl}\right) = \pi_i$$

where $\pi_i$ is the true proportion of success in the $i$th intervention group. Then

$$\mathrm{E}\left(\widehat{\mathrm{RD}}_{\mathrm{unadj}}\right) = \mathrm{E}\left(\bar{p}_1 - \bar{p}_0\right) = \pi_1 - \pi_0 = \mathrm{RD}.$$

Hence $\widehat{\mathrm{RD}}_{\mathrm{unadj}}$ is unbiased for true RD.

Now, since

$$\bar{p}_0 \xrightarrow{prob.} \pi_0 \quad \text{and} \quad \bar{p}_1 \xrightarrow{prob.} \pi_1 \quad \text{as} \quad k \to \infty,$$

$$\widehat{\mathrm{RR}}_{\mathrm{unadj}} = \frac{\bar{p}_1}{\bar{p}_0} \xrightarrow{prob.} \frac{\pi_1}{\pi_0} = \mathrm{RR} \quad \text{as} \quad k \to \infty.$$

Therefore, $\widehat{\mathrm{RR}}_{\mathrm{unadj}}$ is consistent (and, therefore, asymptotically unbiased) for true RR as $k \to \infty$.

## Appendix B

In this appendix, we show that the adjusted cluster-level estimator of risk ratio (RR) with full data is a consistent estimator (and, therefore, asymptotically unbiased) of true RR under certain conditions.

As we defined in equation (5) in the main paper, the adjusted cluster-level estimator of RR is given by

$$\widehat{\mathrm{RR}}_{\mathrm{adj}} = \frac{\bar{\epsilon}_1^r}{\bar{\epsilon}_0^r} = \frac{\frac{1}{k}\sum_{j=1}^{k}\frac{N_{1j}}{\hat{N}_{1j}}}{\frac{1}{k}\sum_{j=1}^{k}\frac{N_{0j}}{\hat{N}_{0j}}} \tag{B1}$$

If $k \to \infty$, the numerator is a consistent estimator of

$$\begin{aligned}
\mathrm{E}\left(\frac{N_{1j}}{\hat{N}_{1j}}\right) &= \mathrm{E}\left[\mathrm{E}\left(\frac{N_{1j}}{\hat{N}_{1j}}\middle|\delta_{1j}, \boldsymbol{X}_{1j}\right)\right] \\
&= \mathrm{E}\left[\frac{\mathrm{E}\left(N_{1j}|\delta_{1j}, \boldsymbol{X}_{1j}\right)}{\hat{N}_{1j}(\boldsymbol{X}_{1j})}\right] \\
&= \mathrm{E}\left[\frac{\sum_{l=1}^{m}\pi_{1jl}}{\hat{N}_{1j}(\boldsymbol{X}_{1j})}\right]
\end{aligned}$$

Assuming the data are generated from the log link model given in equation (1) in the main paper, we have

$$\begin{aligned}
\mathrm{E}\left(\frac{N_{1j}}{\hat{N}_{1j}}\right) &= \mathrm{E}\left[\frac{\sum_{l=1}^{m}\exp\left(\beta_0+\beta_1+f_1(X_{1jl})+\delta_{1j}\right)}{\hat{N}_{1j}(\boldsymbol{X}_{1j})}\right] \\
&= \exp\left(\beta_0+\beta_1\right)\mathrm{E}\left[\frac{\exp\left(\delta_{1j}\right)\sum_{l=1}^{m}\exp\left(f_1(X_{1jl})\right)}{\hat{N}_{1j}(\boldsymbol{X}_{1j})}\right]
\end{aligned} \tag{B2}$$

Similarly, it can be shown that, if $k \to \infty$, the denominator of equation (B1) is a consistent estimator of

$$\begin{aligned}
\mathrm{E}\left(\frac{N_{0j}}{\hat{N}_{0j}}\right) &= \mathrm{E}\left[\frac{\sum_{l=1}^{m}\exp\left(\beta_0+f_0(X_{0jl})+\delta_{0j}\right)}{\hat{N}_{0j}(\boldsymbol{X}_{0j})}\right] \\
&= \exp\left(\beta_0\right)\mathrm{E}\left[\frac{\exp\left(\delta_{0j}\right)\sum_{l=1}^{m}\exp\left(f_0(X_{0jl})\right)}{\hat{N}_{0j}(\boldsymbol{X}_{0j})}\right]
\end{aligned} \tag{B3}$$

The distribution of $X$ (in expectation) is the same between the intervention groups as a consequence of randomisation. If $\delta_{0j}$ and $\delta_{1j}$ have common distribution, and $f_i(X_{ijl}) = f(X_{ijl})$ for $i \in \{0, 1\}$, the expectations in the right hand side of equations (B2) and (B3) are equal. Hence, we have

$$\widehat{\mathrm{RR}}_{\mathrm{adj}} \to \exp(\beta_1) = \mathrm{RR} \qquad \text{as} \qquad k \to \infty.$$

Therefore, the adjusted cluster-level estimator of RR is consistent and, therefore, asymptotically unbiased (as $k \to \infty$) for true RR if (i) the true data generating model is a log link model, (ii) the functional form of the covariates is the same between the intervention groups, and (iii) the distribution of random effect is the same between the intervention groups.

The above argument is not true if the data are generating from the logit link model (2) in the main paper with $\beta_1 \neq 0$, and, therefore, $\widehat{\mathrm{RR}}_{\mathrm{adj}}$ is not consistent for true RR ($\neq 1$). However, under the null hypothesis of no intervention effect ($\beta_1 = 0$), the above argument is true if the true data generating model has logit link. Hence $\widehat{\mathrm{RR}}_{\mathrm{adj}}$ is consistent for true RR ($= 1$) as $k \to \infty$.

## Appendix C

In this appendix we show that the cluster-level analyses for RD using CRA are biased. To this end, we write the individual-level probabilities of success, $\pi_{ijl}$, as

$$\pi_{ijl} = \pi_i + g_i\left(X_{ijl}, \delta_{ij}\right)$$

where $g_i\left(X_{ijl}, \delta_{ij}\right)$ is a function of baseline covariate $X_{ijl}$ and random cluster-effect $\delta_{ij}$, and which determines how individual-level probabilities of success differ from group level probability of success in each intervention group. Then

$$\mathrm{E}_{j,l}\left(\pi_{ijl}|R_{ijl}=1\right) = \pi_i + \mathrm{E}_{j,l}\left(g_i\left(X_{ijl}, \delta_{ij}\right)|R_{ijl}=1\right)$$

and

$$\begin{aligned}
\mathrm{E}\left(\widehat{\mathrm{RD}}^{\mathrm{cr}}_{\mathrm{unadj}}\right) &= \mathrm{E}\left(\pi_{1jl}|R_{1jl}=1\right) - \mathrm{E}\left(\pi_{0jl}|R_{0jl}=1\right) \\
&= \pi_1 - \pi_0 + \mathrm{E}\left(g_1\left(X_{1jl}, \delta_{1j}\right)|R_{1jl}=1\right) - \mathrm{E}\left(g_0\left(X_{0jl}, \delta_{0j}\right)|R_{0jl}=1\right) \\
&= \mathrm{RD} + \mathrm{E}\left(g_1\left(X_{1jl}, \delta_{1j}\right)|R_{1jl}=1\right) - \mathrm{E}\left(g_0\left(X_{0jl}, \delta_{0j}\right)|R_{0jl}=1\right).
\end{aligned}$$

So $\widehat{\mathrm{RD}}^{\mathrm{cr}}_{\mathrm{unadj}}$ will be unbiased for true RD if and only if

$$\mathrm{E}\left(g_1\left(X_{1jl}, \delta_{1j}\right)|R_{1jl}=1\right) = \mathrm{E}\left(g_0\left(X_{0jl}, \delta_{0j}\right)|R_{0jl}=1\right).$$

Assuming the data are generated from the log link model (1) in the main paper, we have

$$g_i\left(X_{ijl}, \delta_{ij}\right) = \pi_{ijl} - \pi_i = \exp(\beta_0 + \beta_1 i)\left\{\exp\left(f_i(X_{ijl}) + \delta_{ij}\right) - \mathrm{E}_{j,l}\left(\exp\left(f_i(X_{ijl}) + \delta_{ij}\right)\right)\right\} \tag{C1}$$

since $\pi_i = \mathrm{E}_{j,l}\left(\pi_{ijl}\right)$. If there is an intervention effect in truth ($\beta_1 \neq 0$), in general, we have from (C1)

$$\mathrm{E}\left(g_1\left(X_{1jl}, \delta_{1j}\right)|R_{1jl}=1\right) \neq \mathrm{E}\left(g_0\left(X_{0jl}, \delta_{0j}\right)|R_{0jl}=1\right)$$

even if the two intervention groups have the same missingness mechanism and the same covariate effects in the data generating model for the outcome. Hence, $\widehat{\mathrm{RD}}^{\mathrm{cr}}_{\mathrm{unadj}}$ is biased for true RD when the true data generating model has log link. However, under the null hypothesis of no intervention effect ($\beta_1 = 0$), if the two intervention groups have the same covariate effect, i.e. $f_i(X_{ijl}) = f(X_{ijl})$ for $i \in \{0, 1\}$, we have

$$g_i\left(X_{ijl}, \delta_{ij}\right) = \exp(\beta_0)\left\{\exp\left(f(X_{ijl}) + \delta_{ij}\right) - \mathrm{E}_{j,l}\left(\exp\left(f(X_{ijl}) + \delta_{ij}\right)\right)\right\}$$

and then, in addition, if the two intervention groups have the same missingness mechanism, we have

$$\mathrm{E}\left(g_1\left(X_{1jl}, \delta_{1j}\right)|R_{1jl}=1\right) = \mathrm{E}\left(g_0\left(X_{0jl}, \delta_{0j}\right)|R_{0jl}=1\right)$$

and hence $\widehat{\mathrm{RD}}^{\mathrm{cr}}_{\mathrm{unadj}}$ is unbiased for true RD $= 0$.

On the other hand, if we assume the data are generated from the logit link model (2) in the main paper, we have

$$
\begin{aligned}
g_i\left(X_{ijl}, \delta_{ij}\right) &= \pi_{ijl} - \pi_i \\
&= \operatorname{expit}\left(\beta_0 + \beta_1 i + f_i(X_{ijl}) + \delta_{ij}\right) - \mathrm{E}_{j,l}\left(\operatorname{expit}\left(\beta_0 + \beta_1 i + f_i(X_{ijl}) + \delta_{ij}\right)\right)
\end{aligned} \tag{C2}
$$

Then, again with $\beta_1 \neq 0$, we have from (C2)

$$
\mathrm{E}\left(g_1\left(X_{1jl}, \delta_{1j}\right) | R_{1jl} = 1\right) \neq \mathrm{E}\left(g_0\left(X_{0jl}, \delta_{0j}\right) | R_{0jl} = 1\right)
$$

even if the two intervention groups have the same missingness mechanism and the same covariate effect. Hence, $\widehat{\mathrm{RD}}^{\mathrm{cr}}_{\mathrm{unadj}}$ is biased for true RD when the true data generating model has logit link. However, like log link, under the null hypothesis of no intervention effect ($\beta_1 = 0$), if the two intervention groups have the same covariate effect, i.e. $f_i(X_{ijl}) = f(X_{ijl})$ for $i \in \{0, 1\}$ and if $\delta_{0j}$ and $\delta_{1j}$ have common distribution, we have

$$
g_i\left(X_{ijl}, \delta_{ij}\right) = \operatorname{expit}\left(\beta_0 + f(X_{ijl}) + \delta_{ij}\right) - \mathrm{E}_{j,l}\left(\operatorname{expit}\left(\beta_0 + f(X_{ijl}) + \delta_{ij}\right)\right)
$$

and then, in addition, if the two intervention groups have the same missingness mechanism, we have

$$
\mathrm{E}\left(g_1\left(X_{1jl}, \delta_{1j}\right) | R_{1jl} = 1\right) = \mathrm{E}\left(g_0\left(X_{0jl}, \delta_{0j}\right) | R_{0jl} = 1\right)
$$

and hence $\widehat{\mathrm{RD}}^{\mathrm{cr}}_{\mathrm{unadj}}$ is unbiased for true RD $= 0$.

# Appendix D

In this appendix we investigate the validity of the cluster-level analyses for RR using CRA. To this end, we write $\pi_{ijl}$ as

$$
\pi_{ijl} = \pi_i \, h_i\left(X_{ijl}, \delta_{ij}\right)
$$

where $h_i\left(X_{ijl}, \delta_{ij}\right)$ is a function of baseline covariate $X_{ijl}$ and random cluster-effect $\delta_{ij}$, and which determines how individual-level probabilities of success differ from group level probability of success. Then

$$
\mathrm{E}_{j,l}\left(\pi_{ijl} | R_{ijl} = 1\right) = \pi_i \, \mathrm{E}_{j,l}\left(h_i\left(X_{ijl}, \delta_{ij}\right) | R_{ijl} = 1\right)
$$

and

$$
\begin{aligned}
\widehat{\mathrm{RR}}^{\mathrm{cr}}_{\mathrm{unadj}} &\longrightarrow \frac{\mathrm{E}\left(\pi_{1jl} | R_{1jl} = 1\right)}{\mathrm{E}\left(\pi_{0jl} | R_{0jl} = 1\right)} \text{ as } k \longrightarrow \infty \\
&= \frac{\pi_1 \, \mathrm{E}\left(h_1\left(X_{1jl}, \delta_{1j}\right) | R_{1jl} = 1\right)}{\pi_0 \, \mathrm{E}\left(h_0\left(X_{0jl}, \delta_{0j}\right) | R_{0jl} = 1\right)} \\
&= \mathrm{RR} \, \frac{\mathrm{E}\left(h_1\left(X_{1jl}, \delta_{1j}\right) | R_{1jl} = 1\right)}{\mathrm{E}\left(h_0\left(X_{0jl}, \delta_{0j}\right) | R_{0jl} = 1\right)}
\end{aligned}
$$

So $\widehat{\mathrm{RR}}^{\mathrm{cr}}_{\mathrm{unadj}}$ will be consistent for true RR if only if

$$\frac{\mathrm{E}\left(h_1\left(X_{1jl},\delta_{1j}\right)|R_{1jl}=1\right)}{\mathrm{E}\left(h_0\left(X_{0jl},\delta_{0j}\right)|R_{0jl}=1\right)}=1.$$

Assuming the data are generated from the log link model (1) in the main paper, we have

$$\begin{aligned} h_i\left(X_{ijl},\delta_{ij}\right) &= \frac{\exp\left(\beta_0+\beta_1 i+f_i(X_{ijl})+\delta_{ij}\right)}{\mathrm{E}_{j,l}\left(\exp\left(\beta_0+\beta_1 i+f_i(X_{ijl})+\delta_{ij}\right)\right)} \\ &= \frac{\exp\left(f_i(X_{ijl})+\delta_{ij}\right)}{\mathrm{E}_{j,l}\left(\exp\left(f_i(X_{ijl})+\delta_{ij}\right)\right)} \end{aligned}$$

and

$$\frac{\mathrm{E}\left(h_1\left(X_{1jl},\delta_{1j}\right)|R_{1jl}=1\right)}{\mathrm{E}\left(h_0\left(X_{0jl},\delta_{0j}\right)|R_{0jl}=1\right)}=\frac{\mathrm{E}\left(\exp\left(f_1(X_{1jl})+\delta_{1j}\right)|R_{1jl}=1\right)}{\mathrm{E}\left(\exp\left(f_0(X_{0jl})+\delta_{0j}\right)|R_{0jl}=1\right)}\times\frac{\mathrm{E}\left(\exp\left(f_0(X_{0jl})+\delta_{0j}\right)\right)}{\mathrm{E}\left(\exp\left(f_1(X_{1jl})+\delta_{1j}\right)\right)}$$

Then if the two intervention groups have the same covariate effect, i.e. $f_i(X_{ijl})=f(X_{ijl})$ for $i\in\{0,1\}$) and if $\delta_{0j}$ and $\delta_{1j}$ have common distribution, we have

$$\frac{\mathrm{E}\left(\exp\left(f_0(X_{0jl})+\delta_{0j}\right)\right)}{\mathrm{E}\left(\exp\left(f_1(X_{1jl})+\delta_{1j}\right)\right)}=1$$

and, in addition, if the two intervention groups have the same missingness mechanism, we have

$$\frac{\mathrm{E}\left(\exp\left(f_1(X_{1jl})+\delta_{1j}\right)|R_{1jl}=1\right)}{\mathrm{E}\left(\exp\left(f_0(X_{0jl})+\delta_{0j}\right)|R_{0jl}=1\right)}=1$$

Therefore, if the two intervention groups have the same missingness mechanism and the same covariate effects, we have

$$\frac{\mathrm{E}\left(h_1\left(X_{1jl},\delta_{1j}\right)|R_{1jl}=1\right)}{\mathrm{E}\left(h_0\left(X_{0jl},\delta_{0j}\right)|R_{0jl}=1\right)}=1$$

and hence $\widehat{\mathrm{RR}}^{\mathrm{cr}}_{\mathrm{unadj}}$ is consistent for true RR.

On the other hand, assuming the data are generated from the logit link model (2) in the main paper, we have

$$h_i\left(X_{ijl},\delta_{ij}\right) = \frac{\mathrm{expit}\left(\beta_0+\beta_1 i+f_i(X_{ijl})+\delta_{ij}\right)}{\mathrm{E}_{j,l}\left(\mathrm{expit}\left(\beta_0+\beta_1 i+f_i(X_{ijl})+\delta_{ij}\right)\right)}$$

and

$$\begin{aligned}\frac{\mathrm{E}\left(h_1\left(X_{1jl},\delta_{1j}\right)|R_{1jl}=1\right)}{\mathrm{E}\left(h_0\left(X_{0jl},\delta_{0j}\right)|R_{0jl}=1\right)} &= \frac{\mathrm{E}\left(\mathrm{expit}\left(\beta_0+\beta_1+f_1(X_{1jl})+\delta_{1j}\right)|R_{1jl}=1\right)}{\mathrm{E}\left(\mathrm{expit}\left(\beta_0+f_0(X_{0jl})+\delta_{0j}\right)|R_{0jl}=1\right)} \\ &\quad\times\frac{\mathrm{E}\left(\mathrm{expit}\left(\beta_0+f_0(X_{0jl})+\delta_{0j}\right)\right)}{\mathrm{E}\left(\mathrm{expit}\left(\beta_0+\beta_1+f_1(X_{1jl})+\delta_{1j}\right)\right)} \end{aligned} \quad \text{(D1)}$$

If $\beta_1\neq 0$, we have

$$\frac{\mathrm{E}\left(\mathrm{expit}\left(\beta_0+f_0(X_{0jl})+\delta_{0j}\right)\right)}{\mathrm{E}\left(\mathrm{expit}\left(\beta_0+\beta_1+f_1(X_{1jl})+\delta_{1j}\right)\right)}\neq 1$$

and

$$\frac{\mathrm{E}\left(\operatorname{expit}\left(\beta_0+\beta_1+f_1(X_{1jl})+\delta_{1j}\right)|R_{1jl}=1\right)}{\mathrm{E}\left(\operatorname{expit}\left(\beta_0+f_0(X_{0jl})+\delta_{0j}\right)|R_{0jl}=1\right)}\neq 1$$

even if the two intervention groups have the same missingness mechanism and the same covariate effects. Hence

$$\frac{\mathrm{E}\left(h_1\left(X_{1jl},\delta_{1j}\right)|R_{1jl}=1\right)}{\mathrm{E}\left(h_0\left(X_{0jl},\delta_{0j}\right)|R_{0jl}=1\right)}\neq 1$$

and therefore $\widehat{\mathrm{RR}}^{\mathrm{cr}}_{\mathrm{unadj}}$ is not consistent for true RR. However, under the null hypothesis of no intervention effect ($\beta_1=0$), if the two intervention group have the same missingness mechanism and the same covariate effect, the both ratios of expectations in the right side of equation (D1) equal to one, and hence we have

$$\frac{\mathrm{E}\left(h_1\left(X_{1jl},\delta_{1j}\right)|R_{1jl}=1\right)}{\mathrm{E}\left(h_0\left(X_{0jl},\delta_{0j}\right)|R_{0jl}=1\right)}=1$$

Therefore, if the data generating model has logit link and there is no intervention effect in truth, $\widehat{\mathrm{RR}}^{\mathrm{cr}}_{\mathrm{unadj}}$ is consistent for true RR $=1$ when the two intervention groups have the same missingness and the same covariate effect.

# Appendix E

As we defined in equation (8), the adjusted cluster-level estimator of RR using complete records is given by

$$\widehat{\mathrm{RR}}^{\mathrm{cr}}_{\mathrm{adj}}=\frac{\bar{\epsilon}_1^{r(\mathrm{cr})}}{\bar{\epsilon}_0^{r(\mathrm{cr})}}=\frac{\frac{1}{k}\sum_{j=1}^{k}\frac{N_{1j}^{\mathrm{cr}}}{\hat{N}_{1j}^{\mathrm{cr}}}}{\frac{1}{k}\sum_{j=1}^{k}\frac{N_{0j}^{\mathrm{cr}}}{\hat{N}_{0j}^{\mathrm{cr}}}} \tag{E1}$$

where $N_{ij}^{\mathrm{cr}}$ and $\hat{N}_{ij}^{\mathrm{cr}}$ are the observed and predicted number of successes for the complete records in the $(ij)$th cluster.

Assuming the data are generated from the log link model (1) in the main paper, and following the similar argument presented in Appendix B, it can be shown that, in the case of CRA, the numerator of equation (E1) is a consistent estimator of

$$\begin{aligned}\mathrm{E}\left(\frac{N_{1j}^{\mathrm{cr}}}{\hat{N}_{1j}^{\mathrm{cr}}}\right)&=\mathrm{E}\left[\frac{\sum_{l=1}^{m}R_{ijl}\exp\left(\beta_0+\beta_1+f_1(X_{1jl})+\delta_{1j}\right)}{\hat{N}_{1j}^{\mathrm{cr}}(\boldsymbol{X}_{1j},\boldsymbol{R}_{1j})}\right]\\&=\exp\left(\beta_0+\beta_1\right)\mathrm{E}\left[\frac{\exp\left(\delta_{1j}\right)\sum_{l=1}^{m}R_{ijl}\exp\left(f_1(X_{1jl})\right)}{\hat{N}_{1j}^{\mathrm{cr}}(\boldsymbol{X}_{1j},\boldsymbol{R}_{1j})}\right],\end{aligned} \tag{E2}$$

and the denominator of equation (E1) is a consistent estimator of

$$\begin{aligned}\mathrm{E}\left(\frac{N_{0j}^{\mathrm{cr}}}{\hat{N}_{0j}^{\mathrm{cr}}}\right)&=\mathrm{E}\left[\frac{\sum_{l=1}^{m}R_{ijl}\exp\left(\beta_0+f_0(X_{0jl})+\delta_{0j}\right)}{\hat{N}_{0j}^{\mathrm{cr}}(\boldsymbol{X}_{0j},\boldsymbol{R}_{0j})}\right]\\&=\exp\left(\beta_0\right)\mathrm{E}\left[\frac{\exp\left(\delta_{0j}\right)\sum_{l=1}^{m}R_{ijl}\exp\left(f_0(X_{0jl})\right)}{\hat{N}_{0j}^{\mathrm{cr}}(\boldsymbol{X}_{0j},\boldsymbol{R}_{0j})}\right],\end{aligned} \tag{E3}$$

where $\boldsymbol{R}_{ij}$ is the vector of missing outcomes indicators of the $(ij)$th cluster. The distribution of $X$ (in expectation) is the same between the intervention groups as a consequence of randomisation. The expectations in the right hand side of equations (E2) and (E3) are equal if $\delta_{0j}$ and $\delta_{1j}$ have common distribution, the missingness mechanism is the same between the intervention groups, and $f_i(X_{ijl}) = f(X_{ijl})$ for $i \in \{0, 1\}$. Hence, we have

$$\widehat{\mathrm{RR}}^{\mathrm{cr}}_{\mathrm{adj}} \to \exp(\beta_1) = \mathrm{RR} \qquad \text{as} \qquad k \to \infty.$$

Therefore, $\widehat{\mathrm{RR}}^{\mathrm{cr}}_{\mathrm{adj}}$ is consistent and, therefore, asymptotically unbiased (as $k \to \infty$) for true RR if (i) the true data generating model is a log link model, (ii) the functional form of the covariates is the same between the intervention groups, (iii) the missingness mechanism is the same between the intervention groups, and (iv) the distribution of random effects is the same between the intervention groups.

The above argument is not true if the data are generated from the logit link model (2) in the main paper with $\beta_1 \neq 0$, and, therefore, $\widehat{\mathrm{RR}}^{\mathrm{cr}}_{\mathrm{adj}}$ is not consistent for true RR ($\neq 1$). However, under the null hypothesis of no intervention effect ($\beta_1 = 0$), the above argument is true if the true data generating model has logit link. Hence $\widehat{\mathrm{RR}}^{\mathrm{cr}}_{\mathrm{adj}}$ is consistent for true RR ($= 1$) as $k \to \infty$.

# Appendix F

Table F1 represents the results of the simulation study, explained in the main paper, for RD using cluster-level analyses with full data, CRA and MMI.

Table F1: Average estimates of RD, their average estimated standard errors (SE) and coverage rates for nominal 95% confidence intervals over 1000 simulation runs, using unadjusted cluster-level ($CL_U$) and adjusted cluster-level ($CL_A$) analyses with full data, CRA and MMI. Monte Carlo errors for average estimates and average estimated SEs are all less than 0.003 and 0.001, respectively. The true value of RD is 20%.

| | $k$ | Average estimate (%) | | | | | | Average estimated SE | | | | | | Coverage (%) | | | | | |
|---|---|---|---|---|---|---|---|---|---|---|---|---|---|---|---|---|---|---|---|
| | | Full | | CRA | | MMI | | Full | | CRA | | MMI | | Full | | CRA | | MMI | |
| | | $CL_U$ | $CL_A$ | $CL_U$ | $CL_A$ | $CL_U$ | $CL_A$ | $CL_U$ | $CL_A$ | $CL_U$ | $CL_A$ | $CL_U$ | $CL_A$ | $CL_U$ | $CL_A$ | $CL_U$ | $CL_A$ | $CL_U$ | $CL_A$ |
| S1 | 5 | 20.0 | 19.9 | 22.7 | 22.5 | 20.2 | 20.1 | 0.069 | 0.051 | 0.074 | 0.061 | 0.074 | 0.058 | 93.8 | 94.3 | 93.4 | 90.3 | 97.3 | 97.1 |
| | 10 | 20.0 | 20.1 | 22.6 | 22.6 | 20.1 | 20.2 | 0.049 | 0.037 | 0.053 | 0.044 | 0.053 | 0.042 | 95.8 | 95.1 | 93.2 | 91.2 | 96.5 | 96.7 |
| | 20 | 20.1 | 20.1 | 22.6 | 22.6 | 20.2 | 20.2 | 0.035 | 0.027 | 0.037 | 0.031 | 0.037 | 0.029 | 95.5 | 94.0 | 89.6 | 86.1 | 95.5 | 95.5 |
| | 50 | 20.0 | 20.0 | 22.6 | 22.6 | 20.1 | 20.1 | 0.022 | 0.017 | 0.024 | 0.020 | 0.023 | 0.018 | 95.1 | 94.8 | 81.5 | 75.5 | 95.2 | 95.5 |
| S2 | 5 | 20.0 | 20.0 | 11.7 | 21.9 | 19.8 | 19.8 | 0.068 | 0.052 | 0.083 | 0.070 | 0.080 | 0.066 | 95.7 | 94.8 | 86.8 | 95.4 | 98.5 | 98.8 |
| | 10 | 20.2 | 20.0 | 12.0 | 21.9 | 20.1 | 19.9 | 0.049 | 0.037 | 0.059 | 0.049 | 0.056 | 0.045 | 96.1 | 95.9 | 74.4 | 94.9 | 97.5 | 97.3 |
| | 20 | 19.9 | 19.9 | 11.7 | 21.9 | 20.0 | 19.9 | 0.035 | 0.027 | 0.042 | 0.036 | 0.039 | 0.032 | 95.0 | 94.5 | 52.2 | 93.0 | 94.9 | 96.2 |
| | 50 | 20.0 | 20.1 | 11.8 | 22.0 | 20.0 | 20.1 | 0.022 | 0.017 | 0.027 | 0.023 | 0.024 | 0.020 | 95.7 | 94.9 | 13.6 | 87.5 | 95.2 | 95.7 |
| S3 | 5 | 20.2 | 20.1 | 19.7 | 19.6 | 20.3 | 20.1 | 0.068 | 0.058 | 0.075 | 0.067 | 0.076 | 0.067 | 93.8 | 94.5 | 93.8 | 94.1 | 96.6 | 97.2 |
| | 10 | 19.9 | 19.9 | 19.6 | 19.6 | 20.0 | 20.0 | 0.050 | 0.042 | 0.055 | 0.048 | 0.055 | 0.047 | 95.7 | 95.9 | 95.7 | 96.1 | 96.3 | 96.8 |
| | 20 | 20.0 | 20.0 | 19.6 | 19.6 | 20.1 | 20.0 | 0.036 | 0.030 | 0.039 | 0.034 | 0.039 | 0.033 | 94.6 | 94.0 | 94.6 | 94.1 | 95.7 | 95.3 |
| | 50 | 20.0 | 20.0 | 19.6 | 19.6 | 20.1 | 20.1 | 0.023 | 0.019 | 0.025 | 0.022 | 0.024 | 0.021 | 95.4 | 95.0 | 95.2 | 94.7 | 95.1 | 94.8 |
| S4 | 5 | 20.3 | 20.2 | 9.2 | 17.4 | 20.0 | 19.9 | 0.071 | 0.058 | 0.085 | 0.076 | 0.086 | 0.075 | 94.7 | 94.0 | 82.3 | 94.4 | 98.6 | 98.8 |
| | 10 | 20.1 | 20.1 | 9.2 | 17.4 | 20.2 | 20.2 | 0.050 | 0.042 | 0.060 | 0.054 | 0.059 | 0.052 | 93.9 | 94.5 | 60.9 | 92.6 | 95.9 | 96.9 |
| | 20 | 19.9 | 20.0 | 8.8 | 17.1 | 19.9 | 20.0 | 0.036 | 0.030 | 0.043 | 0.038 | 0.041 | 0.037 | 95.2 | 94.1 | 29.4 | 89.5 | 95.5 | 96.2 |
| | 50 | 20.0 | 20.0 | 8.8 | 17.1 | 20.0 | 20.0 | 0.023 | 0.019 | 0.027 | 0.024 | 0.026 | 0.023 | 95.0 | 95.7 | 2.3 | 80.0 | 94.8 | 94.4 |

Table F2 presents the results of a further simulation study for cluster-level analyses for RD with full data, CRA and MMI. The parameters configuration was the same with the simulation study explained in the main paper except $\beta_1 = 1$ and, in **(S2)** and **(S4)**, $\beta_{2(0)} = 0.5,\ \beta_{2(1)} = 1$.

Table F2: Further simulation results for RD using cluster-level analyses. Average estimates of RD, their average estimated standard errors (SE) and coverage rates for nominal 95% confidence intervals over 1000 simulation runs, using unadjusted cluster-level ($CL_U$) and adjusted cluster-level ($CL_A$) analyses with full data, CRA and MMI. The true value of RD is 15%.

| | $k$ | Average estimate (%) | | | | | | Average estimated SE | | | | | | Coverage (%) | | | | | |
|---|---|---|---|---|---|---|---|---|---|---|---|---|---|---|---|---|---|---|---|
| | | Full | | CRA | | MMI | | Full | | CRA | | MMI | | Full | | CRA | | MMI | |
| | | $CL_U$ | $CL_A$ | $CL_U$ | $CL_A$ | $CL_U$ | $CL_A$ | $CL_U$ | $CL_A$ | $CL_U$ | $CL_A$ | $CL_U$ | $CL_A$ | $CL_U$ | $CL_A$ | $CL_U$ | $CL_A$ | $CL_U$ | $CL_A$ |
| S1 | 5 | 14.9 | 14.9 | 16.7 | 16.6 | 15.0 | 15.0 | 0.071 | 0.053 | 0.075 | 0.063 | 0.076 | 0.060 | 94.5 | 95.9 | 94.5 | 95.2 | 96.6 | 98.7 |
| | 10 | 15.1 | 15.1 | 16.9 | 16.8 | 15.2 | 15.2 | 0.050 | 0.038 | 0.054 | 0.045 | 0.054 | 0.042 | 94.2 | 93.6 | 93.3 | 92.3 | 95.1 | 94.9 |
| | 20 | 15.1 | 15.0 | 16.8 | 16.7 | 15.2 | 15.1 | 0.036 | 0.027 | 0.038 | 0.032 | 0.038 | 0.030 | 94.5 | 94.4 | 92.8 | 90.8 | 95.3 | 94.5 |
| | 50 | 15.1 | 15.1 | 16.7 | 16.7 | 15.0 | 15.0 | 0.023 | 0.017 | 0.024 | 0.020 | 0.023 | 0.018 | 94.6 | 95.3 | 88.9 | 85.2 | 90.4 | 90.7 |
| S2 | 5 | 14.9 | 14.9 | 5.3 | 15.9 | 14.9 | 15.1 | 0.070 | 0.052 | 0.082 | 0.069 | 0.083 | 0.068 | 94.1 | 95.2 | 82.0 | 95.6 | 97.6 | 98.8 |
| | 10 | 14.9 | 15.1 | 5.5 | 16.0 | 15.1 | 15.0 | 0.050 | 0.038 | 0.059 | 0.050 | 0.058 | 0.048 | 94.9 | 95.1 | 68.6 | 95.1 | 95.5 | 97.0 |
| | 20 | 15.1 | 15.1 | 5.5 | 16.0 | 15.0 | 14.9 | 0.036 | 0.027 | 0.042 | 0.036 | 0.041 | 0.033 | 94.8 | 94.5 | 40.3 | 94.5 | 95.4 | 95.8 |
| | 50 | 15.0 | 15.0 | 5.5 | 16.0 | 15.0 | 15.0 | 0.023 | 0.017 | 0.027 | 0.023 | 0.025 | 0.021 | 94.6 | 94.3 | 6.0 | 94.2 | 95.8 | 96.8 |
| S3 | 5 | 15.2 | 15.2 | 13.2 | 13.2 | 15.5 | 15.4 | 0.072 | 0.061 | 0.078 | 0.070 | 0.081 | 0.071 | 95.6 | 96.4 | 93.7 | 94.1 | 97.6 | 99.1 |
| | 10 | 15.0 | 15.0 | 12.9 | 12.9 | 15.0 | 15.1 | 0.052 | 0.044 | 0.056 | 0.050 | 0.057 | 0.050 | 94.8 | 94.8 | 93.5 | 93.8 | 96.8 | 96.8 |
| | 20 | 15.0 | 15.0 | 13.0 | 12.9 | 15.1 | 15.1 | 0.036 | 0.031 | 0.039 | 0.035 | 0.040 | 0.035 | 94.3 | 93.9 | 92.7 | 90.0 | 95.6 | 95.4 |
| | 50 | 15.1 | 15.2 | 13.0 | 13.0 | 15.1 | 15.2 | 0.023 | 0.020 | 0.025 | 0.023 | 0.025 | 0.022 | 94.7 | 96.2 | 85.4 | 86.5 | 94.6 | 95.4 |
| S4 | 5 | 15.1 | 14.9 | 1.8 | 9.5 | 15.0 | 14.8 | 0.072 | 0.061 | 0.084 | 0.076 | 0.089 | 0.080 | 96.0 | 95.5 | 73.8 | 92.8 | 98.7 | 99.0 |
| | 10 | 15.1 | 15.1 | 1.9 | 9.8 | 15.1 | 15.0 | 0.051 | 0.044 | 0.061 | 0.055 | 0.062 | 0.056 | 93.6 | 94.0 | 45.6 | 86.3 | 96.6 | 97.1 |
| | 20 | 15.1 | 15.0 | 1.7 | 9.7 | 15.1 | 15.0 | 0.036 | 0.031 | 0.043 | 0.039 | 0.043 | 0.039 | 94.4 | 96.0 | 15.8 | 72.2 | 96.1 | 97.0 |
| | 50 | 15.0 | 15.0 | 1.8 | 9.8 | 15.1 | 15.1 | 0.023 | 0.020 | 0.027 | 0.025 | 0.027 | 0.024 | 94.6 | 94.4 | 0.3 | 42.6 | 95.2 | 95.1 |

Table F3 shows the results of the simulation study, explained in the main paper, for RR using cluster-level analyses with full data, CRA and MMI.

Table F3: Average estimates of log(RR), their average estimated standard errors (SE) and coverage rates for nominal 95% confidence intervals over 1000 simulation runs, using unadjusted cluster-level ($CL_U$) and adjusted cluster-level ($CL_A$) analyses with full data, CRA and MMI. Monte Carlo errors for average estimates and average estimated SEs are all less than 0.005 and 0.001, respectively. The true value of log(RR) is 0.34.

| | $k$ | Average estimate | | | | | | Average estimated SE | | | | | | Coverage (%) | | | | | |
|---|---|---|---|---|---|---|---|---|---|---|---|---|---|---|---|---|---|---|---|
| | | Full | | CRA | | MMI | | Full | | CRA | | MMI | | Full | | CRA | | MMI | |
| | | $CL_U$ | $CL_A$ | $CL_U$ | $CL_A$ | $CL_U$ | $CL_A$ | $CL_U$ | $CL_A$ | $CL_U$ | $CL_A$ | $CL_U$ | $CL_A$ | $CL_U$ | $CL_A$ | $CL_U$ | $CL_A$ | $CL_U$ | $CL_A$ |
| S1 | 5 | 0.339 | 0.344 | 0.461 | 0.464 | 0.344 | 0.348 | 0.123 | 0.096 | 0.159 | 0.136 | 0.135 | 0.110 | 94.4 | 93.9 | 90.5 | 86.2 | 97.3 | 98.0 |
| | 10 | 0.338 | 0.345 | 0.456 | 0.464 | 0.340 | 0.348 | 0.087 | 0.069 | 0.114 | 0.098 | 0.094 | 0.078 | 95.0 | 94.8 | 85.7 | 78.1 | 96.3 | 97.4 |
| | 20 | 0.339 | 0.345 | 0.456 | 0.464 | 0.341 | 0.348 | 0.062 | 0.049 | 0.080 | 0.069 | 0.066 | 0.054 | 94.7 | 93.4 | 71.6 | 58.8 | 95.6 | 95.3 |
| | 50 | 0.336 | 0.343 | 0.453 | 0.461 | 0.339 | 0.346 | 0.039 | 0.031 | 0.051 | 0.044 | 0.041 | 0.034 | 95.5 | 95.1 | 38.2 | 18.4 | 95.7 | 95.5 |
| S2 | 5 | 0.339 | 0.346 | 0.261 | 0.515 | 0.338 | 0.344 | 0.122 | 0.096 | 0.186 | 0.161 | 0.142 | 0.119 | 95.8 | 94.7 | 94.0 | 85.8 | 98.7 | 98.8 |
| | 10 | 0.341 | 0.344 | 0.266 | 0.514 | 0.340 | 0.343 | 0.087 | 0.069 | 0.130 | 0.112 | 0.098 | 0.082 | 95.7 | 95.8 | 92.2 | 69.1 | 97.5 | 97.8 |
| | 20 | 0.336 | 0.342 | 0.260 | 0.512 | 0.337 | 0.343 | 0.062 | 0.049 | 0.093 | 0.081 | 0.069 | 0.057 | 95.4 | 94.6 | 88.0 | 45.4 | 95.4 | 96.2 |
| | 50 | 0.337 | 0.345 | 0.263 | 0.516 | 0.337 | 0.346 | 0.039 | 0.031 | 0.059 | 0.052 | 0.043 | 0.036 | 95.7 | 94.3 | 76.2 | 6.1 | 96.7 | 95.2 |
| S3 | 5 | 0.343 | 0.342 | 0.388 | 0.387 | 0.347 | 0.346 | 0.123 | 0.107 | 0.155 | 0.141 | 0.140 | 0.126 | 93.9 | 93.6 | 94.0 | 93.8 | 96.7 | 96.8 |
| | 10 | 0.336 | 0.338 | 0.383 | 0.383 | 0.338 | 0.340 | 0.089 | 0.077 | 0.112 | 0.102 | 0.099 | 0.088 | 95.7 | 95.8 | 93.6 | 94.3 | 96.5 | 96.9 |
| | 20 | 0.338 | 0.339 | 0.382 | 0.382 | 0.339 | 0.340 | 0.064 | 0.055 | 0.080 | 0.073 | 0.070 | 0.062 | 94.8 | 94.3 | 91.4 | 90.1 | 96.0 | 95.2 |
| | 50 | 0.337 | 0.339 | 0.383 | 0.384 | 0.339 | 0.341 | 0.040 | 0.035 | 0.051 | 0.046 | 0.044 | 0.039 | 95.6 | 95.2 | 86.5 | 84.2 | 95.8 | 95.7 |
| S4 | 5 | 0.347 | 0.346 | 0.200 | 0.385 | 0.342 | 0.341 | 0.128 | 0.109 | 0.186 | 0.167 | 0.154 | 0.138 | 94.2 | 94.2 | 91.8 | 94.6 | 98.9 | 99.0 |
| | 10 | 0.340 | 0.342 | 0.198 | 0.385 | 0.342 | 0.344 | 0.089 | 0.078 | 0.130 | 0.118 | 0.105 | 0.095 | 93.5 | 94.0 | 81.8 | 93.4 | 95.8 | 97.2 |
| | 20 | 0.336 | 0.340 | 0.189 | 0.377 | 0.336 | 0.339 | 0.063 | 0.055 | 0.092 | 0.084 | 0.073 | 0.066 | 95.3 | 94.2 | 66.2 | 92.6 | 95.4 | 95.6 |
| | 50 | 0.336 | 0.340 | 0.189 | 0.376 | 0.338 | 0.340 | 0.040 | 0.035 | 0.058 | 0.053 | 0.045 | 0.041 | 94.8 | 96.0 | 30.4 | 89.3 | 94.8 | 95.4 |

Table F4 shows the results of a further simulation study for adjusted cluster-level analysis for RR with full data. The parameters configuration was the same with the simulation study explained in the main paper except the variance components parameters for generating the baseline covariate $X$. We set $\sigma_u^2 = 0.35$, $\sigma_\alpha^2 = 3.20$ and thus we had $\sigma_x^2 = 3.55, \rho_x = 0.9$.

Table F4: Further simulation results for adjusted cluster-level analysis for RR with full data. Average estimates of log(RR), their empirical standard errors (SE), their average estimated SE, and coverage rates for nominal 95% confidence intervals over 1000 simulation runs, using unadjusted cluster-level ($CL_U$) and adjusted cluster-level ($CL_A$) analyses with full data. The true value of log(RR) is 0.34.

| $k$ | Average estimate | | Empirical SE | | Average estimated SE | | Coverage (%) | |
|---|---|---|---|---|---|---|---|---|
| | $CL_U$ | $CL_A$ | $CL_U$ | $CL_A$ | $CL_U$ | $CL_A$ | $CL_U$ | $CL_A$ |
| 5 | 0.341 | 0.439 | 0.336 | 0.185 | 0.343 | 0.208 | 96.2 | 94.8 |
| 10 | 0.342 | 0.460 | 0.230 | 0.135 | 0.234 | 0.146 | 95.9 | 91.4 |
| 20 | 0.341 | 0.468 | 0.160 | 0.097 | 0.160 | 0.100 | 95.7 | 78.4 |
| 50 | 0.339 | 0.476 | 0.100 | 0.062 | 0.101 | 0.063 | 95.3 | 38.8 |
| 100 | 0.338 | 0.477 | 0.070 | 0.043 | 0.070 | 0.045 | 95.1 | 9.4 |

Table F5 represents the association of the baseline covariates (age, sex, exam score, literacy group and baseline anaemia) of the HALI trial with anaemia at 24 months and with the probability of anaemia outcome at 24 months being missing.

Table F5: Estimates of log odds ratios as measures of association of the baseline covariates with anaemia at 24 months and with the probability of anaemia outcome at 24 months being missing

| | Anaemia | | | Missingness of anaemia | | |
|---|---|---|---|---|---|---|
| | Estimate | Std. Error | p-value | Estimate | Std. Error | p-value |
| Intercept | -1.72 | 0.81 | 0.03 | -2.10 | 0.60 | 0.00 |
| IST (intervention) | 0.36 | 1.10 | 0.74 | -0.27 | 0.83 | 0.74 |
| Age (years) | 0.07 | 0.02 | $< 0.001$ | 0.06 | 0.02 | $< 0.001$ |
| Sex (male vs female) | -0.04 | 0.10 | 0.73 | -0.08 | 0.11 | 0.48 |
| Exam score | 0.00 | 0.00 | 0.77 | 0.00 | 0.00 | 0.91 |
| Literacy group | 0.06 | 0.19 | 0.74 | -0.28 | 0.13 | 0.03 |
| Baseline anaemia | 1.57 | 0.11 | $< 0.001$ | 0.09 | 0.11 | 0.42 |
| IST: Age * | 0.01 | 0.03 | 0.62 | 0.04 | 0.03 | 0.12 |
| IST: Sex * | 0.10 | 0.14 | 0.49 | -0.18 | 0.15 | 0.24 |
| IST: Exam score * | 0.00 | 0.00 | 0.59 | 0.00 | 0.00 | 0.62 |
| IST: Literacy group * | 0.37 | 0.26 | 0.15 | 0.38 | 0.19 | 0.04 |
| IST: Baseline anaemia * | -0.19 | 0.15 | 0.19 | -0.03 | 0.15 | 0.86 |

* Interaction terms
